# *Ildr1* gene deletion protects against diet-induced obesity and hyperglycemia

**DOI:** 10.1371/journal.pone.0270329

**Published:** 2022-06-24

**Authors:** Rashmi Chandra, Dipendra K. Aryal, Jonathan D. Douros, Rafiq Shahid, Supriya J. Davis, Jonathan E. Campbell, Olga Ilkayeya, Phillip J. White, Ramona Rodriguez, Christopher B. Newgard, William C. Wetsel, Rodger A. Liddle

**Affiliations:** 1 Department of Medicine, Division of Gastroenterology, Duke University Medical Center, Durham, North Carolina, United States of America; 2 Department of Psychiatry and Behavioral Sciences, Duke University Medical Center, Durham, North Carolina, United States of America; 3 Duke Molecular Physiology Institute, Duke University, Durham, North Carolina, United States of America; 4 Swarthmore College, Swarthmore, Pennsylvania, United States of America; 5 Sarah W. Stedman Nutrition and Metabolism Center, Duke University, Durham, North Carolina, United States of America; 6 Mouse Behavioral and Neuroendocrine Analysis Core Facility, Duke University, Durham, North Carolina, United States of America; 7 Department of Cell Biology, Duke University Medical Center, Durham, North Carolina, United States of America; 8 Department of Neurobiology, Duke University Medical Center, Durham, North Carolina, United States of America; 9 Department of Veterans Affairs Medical Center, Durham, North Carolina, United States of America; Medical University of Vienna, AUSTRIA

## Abstract

**Objective:**

Immunoglobulin-like Domain-Containing Receptor 1 (ILDR1) is expressed on nutrient sensing cholecystokinin-positive enteroendocrine cells of the gastrointestinal tract and it has the unique ability to induce fat-mediated CCK secretion. However, the role of ILDR1 in CCK-mediated regulation of satiety is unknown. In this study, we examined the effects of ILDR1 on food intake and metabolic activity using mice with genetically-deleted *Ildr1*.

**Methods:**

The expression of ILDR1 in murine tissues and the measurement of adipocyte cell size were evaluated by light and fluorescence confocal microscopy. The effects of *Ildr1* deletion on mouse metabolism were quantitated using CLAMS chambers and by targeted metabolomics assays of multiple tissues. Hormone levels were measured by ELISA. The effects of *Ildr1* gene deletion on glucose and insulin levels were determined using *in vivo* oral glucose tolerance, meal tolerance, and insulin tolerance tests, as well as *ex vivo* islet perifusion.

**Results:**

ILDR1 is expressed in a wide range of tissues. Analysis of metabolic data revealed that although *Ildr1*^*-/-*^ mice consumed more food than wild-type littermates, they gained less weight on a high fat diet and exhibited increased metabolic activity. Adipocytes in *Ildr1*^*-/-*^ mice were significantly smaller than in wild-type mice fed either low or high fat diets. ILDR1 was expressed in both alpha and beta cells of pancreatic islets. Based on oral glucose and mixed meal tolerance tests, *Ildr1*^*-/-*^ mice were more effective at lowering post-prandial glucose levels, had improved insulin sensitivity, and glucose-regulated insulin secretion was enhanced in mice lacking ILDR1.

**Conclusion:**

*Ildr1* loss significantly modified metabolic activity in these mutant mice. While *Ildr1* gene deletion increased high fat food intake, it reduced weight gain and improved glucose tolerance. These findings indicate that ILDR1 modulates metabolic responses to feeding in mice.

## Introduction

Immunoglobulin-like domain-containing receptor 1 (ILDR1) was first identified in a screen for genes likely to be involved in the progression of non-Hodgkin’s lymphoma [1]. Since its discovery, ILDR1 has been found to be necessary for a number of important physiologic actions including regulation of fat-mediated cholecystokinin (CCK) secretion [2, 3], maintenance of water homeostasis in the kidney [4], audition [5–7], and the regulation of alternative RNA splicing [8]. ILDR1 shares approximately 30% amino acid homology with two other proteins, lipolysis-stimulated receptor (LSR) and ILDR2. LSR has previously been shown to play a role in clearance of lipids by removing apo-B containing lipoproteins in conjunction with the LDL receptor [9]. Recently, increased expression of nuclear LSR was shown to be associated with poor outcomes in breast cancer [10], while an anti-ILDR2 antibody promoted T cell activation leading to anti-tumor effects [11]. Thus, the ILDR family of proteins displays multifunctional and diverse roles in mammalian tissues.

Given the effects of ILDR1 on fat-mediated CCK secretion, we examined whether the global loss of ILDR1 would affect food intake in mice. CCK is a peptide hormone that stimulates pancreatic exocrine secretion, gastric motility, and gallbladder contraction [12, 13]. CCK is released from discrete enteroendocrine cells (EECs) located in the intestinal mucosa following ingestion of fats or amino acids [14–19]. Within a few minutes of food intake, plasma levels of CCK rise from basal levels of 0.5–1 pM to peak levels of 5–15 pM [20]. CCK released from EECs binds to CCK1 receptors located on vagal afferent fibers which relay signals to the hypothalamus leading to the feeling of fullness and the arrestment of hunger [21–23]. Hence, CCK plays an important role in the regulation of food intake.

In the present studies we analyze the effects of *Ildr1* gene deletion on food intake and glucose regulation in mice. We demonstrate that in spite of eating more of a diet high in fat than their wild-type littermates, *Ildr1*^*-/-*^ mice fail to gain as much body weight due to a decreased accumulation of fat. In addition, *Ildr1* gene deletion leads to reduced plasma glucose levels as a result of improved insulin sensitivity and secretion.

## Materials and methods

### Transgenic and knockout mice

Swiss Webster CCK-EGFP transgenic mice were procured from the Mutant Mouse Resource and Research Center (MMRRC; University of Missouri, Columbia, MO) and the colony was by propagated by mating CCK-EGFP transgenic mice with wild-type Swiss Webster mice (Charles River Laboratories International, Inc. Wilmington, MA) at each generation [24]. *Ildr1*^*-/-*^ mice were generated at the Duke Neurotransgenics Facility using the gene-trapped ES cell clone D178D03 from the TBV-2 cell line (Helmholtz Zentrum Munchen, Neuherberg, Germany) as described previously [2]. The *Ildr1*^*-/-*^ mice (on a Swiss Webster genetic background) were mated to CCK-EGFP transgenic mice [24] to create *Ildr1*^*-/-*^*;Cck-EGFP* mice (termed *Ildr1*^*-/-*^ mice). Heterozygous *Ildr*^*+/-*^ mice were mated to generate *Ildr1*^*-/-*^ and *Ildr1*^*+/+*^ littermates for experiments. All procedures and behavioral tests were conducted in accordance with protocols approved by the Duke University Animal Care and Use Committee. This mouse strain has been deposited and is available from the MMRRC (RRID: MMRRC_068066-JAX) with the official strain name (stock *Ildr1*^*Gt(D178D03)Wrst*^/RaliMmjax).

### Expression of β-galactosidase reporter in *Ildr1*^*-/-*^ mice

Wild type and *Ildr1*^*-/-*^ mice were transcardially perfused with ice-cold heparinized PBS, followed by freshly depolymerized 3.5% paraformaldehyde in PBS. Tissues were dissected and post-fixed in 3.5% paraformaldehyde, cryopreserved sequentially in 10%, 20%, and 30% sucrose in 0.1M phosphate buffer, pH 7.4, embedded in Tissue-Tek® OCT compound (VWR, Radnor, PA), and cryosectioned at 8–10 μm. Sections were post-fixed in 0.2% glutaraldehyde, 5 mM EGTA in PBS for 10 min at 4°C, stained with 5-bromo-4-chloro-3-indolyl-β-D-galactopyranoside (X-gal) using the protocol developed by Kishigami and colleagues [25]. After X-gal color development, sections were rinsed and counterstained with Nuclear Fast Red stain (Millipore Sigma, St. Louis, MO) for 10 min, followed by two washes in PBS. Slides were dehydrated by passing them sequentially through 70%, 95%, and 100% ethanol (2 times, 30 sec each), they were cleared in xylene (twice, 5 min each) and mounted in Krystalon (Millipore Sigma).

### Metabolic data collection

Wild-type and *Ildr1*^*-/-*^ male mice (ages 6–7 weeks) were placed into CLAMS chambers (Columbus Instruments, Columbus, OH) for 10 days at 73°F (22.7°C). The CLAMS apparatus included 8 chambers. Four wild-type and four *Ildr1*^*-/-*^ mice were placed into CLAMS chambers at the same time, and 3 runs were performed to accommodate all mice (12 wild-type and 12 *Ildr1*^*-/-*^ mice). To minimize chamber position bias, the four chambers that housed wild-type mice in the first run, were used to house *Ildr1*^*-/-*^ mice in the second run. In the third run, use of chambers was mixed again. Each cohort of mice were provided crushed Rodent 5001 (13.6 kcal % fat; LabDiet, St. Louis, MO) for 5 days followed by crushed D12492 irradiated (60 kcal % fat diet; Research Diets, Inc., New Brunswick, NJ) for the next 5 days. Feeding and drinking behavior, locomotor activity, energy expenditure, O_2_ intake, and CO_2_ output, were monitored with Oxymax software as described [26]. Data collected from the first two days with either diet were discarded due to habituation to the chambers and diets. After collection of data from CLAMS, mice were maintained in their home cages either on irradiated 5053 Picolab Rodent Diet 20 (13.12 kcal % fat; LabDiet) or D12492 irradiated chow for an additional 18 days. After blood collection from the tail vein for measurement of glucose, mice were euthanized by decapitation. Blood was collected from the trunk and used for measurement of serum cholesterol (#439–17501, FUJIFILM Wako Diagnostics, Mountain View, CA) and serum triglycerides (#10010303, Cayman Chemicals, Ann Arbor, MI).

### Adipose tissue and adipocyte measurements

After weaning, groups comprised of age-matched wild-type or *Ildr1*^*-/-*^ littermates (n = 3–4 mice/group) were placed on 5001 low fat (13.6 kcal% fat; LabDiet) or D12492 (60% fat; Research Diet) for 4 weeks. After collection of adipocyte tissues the ratio of adipose tissue to body weight was calculated. Retroperitoneal and inguinal adipose tissues (n = 3–4 mice/group) were embedded in paraffin, 5 μm sections were cut, and stained with H&E. Images were collected and adipocyte size (using >500 cells/mouse) was measured using NIH Image J software.

### Plasma hormone measurement

After weaning, age-matched wild-type or *Ildr1*^*-/-*^ littermates (n = 4–6 mice/group) were placed on 5001 13.5% fat chow (LabDiet) or D12492 60% fat diet (Research Diet) for 4 weeks as described above. Plasma hormone levels were measured by ELISA as described by the manufacturer: leptin (#A05176, Bertin Corporation, Rockville, MD), adiponectin (#A05187, Bertin Corporation, Rockville, MD), and T4 (#T4044T-100, Calbiotech, El Cajon, CA).

### RNA isolation

White or brown fat was dissected from the mice (used in hormone assays above) and RNA was isolated using the RNeasy Plus Universal Mini kit (Qiagen, Valencia, CA) following the manufacturer’s recommendations. RNA was examined on a denaturing gel to assess integrity prior to reverse transcription.

### Quantitative real time PCR

Four micrograms of total RNA were reverse transcribed using MultiScribe^TM^ reverse transcriptase kit (Life Technologies, Grand Island, NY). The cDNA was diluted 10-fold and used for quantitative real-time PCR with Taqman gene expression assays (Applied Biosystems, Foster City, CA) on an Applied Biosystems QPCR System. Amplification was performed using the following cycle: 50°C for 2 min (1 cycle); 95°C for 10 min (1 cycle); 95°C for 15 seconds and 60°C for 1 min (40 cycles). The assay IDs of inventoried mouse Taqman gene expression assays were as follows: adiponectin (Mm00456425_m1), β-actin (Mm00607939_s1), Fasn (Mm00662319_m1), Ildr1 (Mm00506487_m1), leptin (Mm00434759_m1), Mest (Mm00485003_m1), Pparg (Mm01184322_m1), Prdm16 (Mm00712556_m1), Tnfa (Mm00443260_g1), and Ucp1 (Mm01244861_m1). The mRNA expression values were compared using the ΔΔC_t_ calculations. The expression of target genes in wild-type tissues was compared to those from *Ildr1*^*-/-*^mice. The data were normalized to *Actb* mRNA levels.

### ILDR antibody development

Mouse *Ildr1* (accession number BC057644, 1317–1650 bp) and human *ILDR1* (accession number AY672837, bp 1318–1674) were cloned downstream of maltose binding protein (MBP) in the pMAL vector (New England Biolabs, Ipswitch, MA) which had been modified to include an in-frame PreScission protease cleavage site between MBP and multiple cloning site (MCS) linker (*Bam*H1 and *Xho*I restriction enzyme sites were used for cloning mouse and human cDNAs), and a 6X histidine tag at the 3’ end of the MCS (gift from Jonathan Q. Davis, Howard Hughes Medical Institute, Duke University, Durham, NC 27710). The clones were expressed using BL21(DE3)pLysS *E*. *coli strain*. Bacteria were induced at A600 nm = 0.5 optical density with 0.4 mM isopropyl β-D-1-thiogalactopyranoside (IPTG) and subsequently grown at room temperature for 4.5 hrs. The bacterial culture was centrifuged (7,000 x g for 10 min at 4°C), the pelleted cells were washed with column buffer (20 mM Tris-HCl, pH 7.4, 1 mM EDTA, 0.2 M NaCl, 1 mM NaN_3_, 1 mM DTT), and frozen at -80°C until use. Protein was extracted by suspending the bacteria in 50 mL of column buffer containing protease inhibitors (cOmplete^TM^, EDTA-free Protease Inhibitor Cocktail, Millipore Sigma, Burlington, MA), sonicating the suspension on ice with 10 sec pulses to shear genomic DNA followed by centrifugation (24,700 x g for 30 min at 4°C). The lysate was filtered (0.22 μm filter) and loaded onto an amylose column (New England Biolabs). The amylose column was washed with five volumes of column buffer and eluted with 10 mM maltose in column buffer. Fractions of 1.5 mL were collected manually and analyzed by SDS-PAGE gel electrophoresis. Fractions containing the fusion protein were pooled and incubated with PreScission protease (gift from Jonathan Q. Davis) overnight on ice. The digest was centrifuged at 29,400 x g for 45 min at 4°C (Ti70 ultracentrifuge rotor, Beckman Instruments, Indianapolis, IN). The supernatant was adjusted to 20 mM sodium phosphate, pH 7.4, 0.5 M NaCl, 40 mM imidazole (binding buffer), and loaded onto a Ni^2+^ Sepharose High Performance column (#17-5268-01; Amersham Biosciences, Buckinghamshire, U.K.). The column was washed extensively with binding buffer and ILDR1 protein was eluted with 0.5 M imidazole in binding buffer. The purity of the eluted ILDR1 protein was assessed by SDS-PAGE gel electrophoresis. The protein was dialyzed and concentrated, and a mixture of mouse and human ILDR1 proteins was used to immunize rabbits (PrimmBiotech Inc., Cambridge MA). The antiserum was affinity purified by passing sequentially over MBP, human, and mouse ILDR1 protein Sepharose columns (GE Healthcare, Chicago, IL) and the quality of the antibody was confirmed by immunoblotting.

### Immunocytochemistry

Mice tissues were harvested with or without transcardial perfusion using 3.5% freshly depolymerized paraformaldehyde, post-fixed for 1 hr in 3.5% paraformaldehyde, and embedded in OCT (VWR, Radnor, PA). Cryosections (5–20 μm) were collected on plus-charged slides, and post-fixed in 10% neutral buffered formalin or in a 1:1 chilled mixture of methanol and acetone for 10 min. Human duodenal tissue specimens were obtained from Duke BioRepository and Precision Pathology Center (BRPC), a shared resource of the Duke University School of Medicine and Duke Cancer Institute. The BRPC operates under the Duke University Institutional Review Board protocol number Pro00035974, under which it can consent patients and distribute de-identified/linked specimens or it can distribute fully anonymized biospecimens under waiver of consent in accordance with federal, state, and local regulations. In this study, fully anonymized specimens were used under a waiver of consent. After surgical excision, deidentified human tissue was immediately fixed in 10% neutral buffered formalin for 24 hrs, cryopreserved in 30% sucrose, frozen in OCT, and cryosectioned at 10 μm. Sections were permeabilized in Tris-buffered saline (TBS; 10 mM Tris, pH 7.4, 0.9% NaCl) containing 0.5% Triton X-100. The following primary antibodies were used: polyclonal chicken anti-β-galactosidase (# ab9361, RRID:AB_307210, 1:100 dilution; abcam, Boston, MA,), polyclonal rabbit anti-ILDR1 (described above, 1:100 dilution), polyclonal guinea pig anti-insulin (# IR002, RRID:AB_2800361; 1:500 dilution; Agilent, Santa Clara, CA; or # PA1-26938, RRID:AB_794668, 1:50 dilution; Thermo Fisher Scientific, Waltham, MA), polyclonal rabbit anti-glucagon (Agilent Cat# A0565, RRID:AB_10013726, 1:500 dilution; Thermo Fisher Scientific, Waltham, MA). Tyramide amplification was performed for the ILDR1 antibody as described by the manufacturer (ThermoFisher Scientific, Waltham, MA). Cross-adsorbed secondary antibodies (Jackson ImmunoResearch, West Grove, PA) were used at 1:250–1:500 dilution. Immunostained sections were mounted in ProLong Gold (ThermoFisher Scientific) and confocal microscopy was performed using a Zeiss 780 or 880 (airy scan) confocal microscope. The 3D images were rendered from Z-stacks using Imaris software (Bitplane, Inc. Concord, MA).

### Glucose assessments

Assessments were conducted with mice maintained on a low fat diet (13.6 kcal % fat, Lab Diet 5001). An oral glucose tolerance test (OGTT) was performed on low fat diet fed wild-type and *Ildr1*^*-/-*^ fasted (18 hours) mice (n = 5 mice/group) as described previously [27]. Mice were gavaged with 2 g/kg glucose and glucose levels were measured with a Contour glucometer (Ascensia Diabetes Care Holdings AG, Basel, Switzerland) at 0, 15, 30, 60, 120, and 180 min using venous tail blood.

*In vivo* assessments of glucose control by mixed meal tolerance test (MMTT) and insulin tolerance test (ITT) were performed as described previously [28]. The MMTT was performed by fasting mice for 5 hr (0800–1300 hr), and then administering 200 μL Ensure Plus by oral gavage. Blood glucose was measured from the tail vein at t = 0, 10, 20, 30, 60, 90, and 120 min using a Bayer glucometer (Leverkusen, Germany), and blood samples (~25 μL) were collected at 0 and 10 min in EDTA-coated tubes (#NC9299309; Sarstedt Microvette, ThermoFisher Scientific). ITTs were performed by fasting mice for 5 hr (0800–1300 hr), then delivering an insulin dose of 0.5 U/kg (Humalog, Eli Lilly, Indianapolis, IN). Blood glucose was measured at 0, 10, 20, 30, and 60min.

*Ex vivo* assessment of islet function: Protocols for *ex vivo* islet perifusion have been reported previously [29]. Isolation of primary mouse islets was achieved by inflating the pancreas through the pancreatic duct with type V collagenase (0.8 mg/ml, # C5138, Millipore Sigma). The inflated pancreas was then excised, and digested for 12 min at 37°C, washed with ice-cold RPMI medium [10 mM glucose (Millipore Sigma), 2 mM L-glutamine (Millipore Sigma), 100 U/ml penicillin (ThermoFisher), 0.25% BSA (Sigma), and 100 μg/ml streptomycin (ThermoFisher)], before separating the islets using a standard Histopaque gradient. Islets were allowed to recover overnight in RPMI medium with 10% FBS at 37°C for further *ex vivo* perifusion experiments. Islet perifusion experiments were performed the following day using the Biorep Perifusion apparatus (Miami, FL) with 75 islets per chamber. All perifusion experiments were conducted in a base KRPH buffer (135 mM NaCl, 3.6 mM KCl, 1.5 mM CaCl_2_, 0.5 mM NaH_2_PO_4_, 0.5 mM MgSO_4_, 5 mM HEPES, 5 mM NaCO_3_, 0.1% BSA, pH 7.5), with a 200 μL/min flow rate, which was preceded by a 48-min equilibration period with KRPH plus 2 mM glucose. Islets from wild-type (*n* = 6) and *Ildr1*^*-/-*^ (*n* = 6) mice were exposed to 2 mM glucose for 8 min, followed by 16 min of 10 mM glucose. Islets were then exposed to 2 mM glucose to bring the insulin secretion back to baseline, followed by treatment with 2 mM glucose and 30 mM KCl. All samples were stored at –20°C before being assayed for insulin.

Peptide assays: Plasma was collected by centrifugation (1000 x *g*, for 15 min) at 4°C and stored at -20°C. Circulating insulin was assayed using an ultra-sensitive mouse insulin ELISA (#90080; CrystalChem, Inc., Elk Grove Village, IL). Perifusion samples were assayed for insulin using the Perkin-Elmer (Waltham, MA) alphaLISA kit (#AL-204) according to manufacturer’s instructions.

### Determination of metabolomics profile

One drop of blood was collected from the tail vein on Whatman 903 filter paper prior to euthanization. Tissues (epididymal fat, blood, liver, gastrocnemius muscle,) were harvested from mice and frozen in liquid nitrogen. The tissues were pulverized in liquid nitrogen, between 70–90 mg powder was added to tubes and extracted for 2 min in a Qialyzer (Qiagen, Germantown, MD) with a mixture of ice-cold 50% acetonitrile and 0.3% formic acid. The extract was centrifuged at 8,000 x g for 5 min at room temperature. Amino acid, acylcarnitine, and ceramide levels were measured by tandem mass spectrometry (MS/MS) methods, as previously described [30, 31]. All MS analyses employed stable-isotope-dilution with internal standards from Isotec (Canton, GA), Cambridge Isotopes Laboratories, (Tewksbury, MA), and CDN Isotopes (Pointe-Claire, Quebec, Canada).

### Statistics

The results are expressed as mean ± SEM. Statistical differences between wild-type and *Ildr1*^*-/-*^ genotypes were examined by the independent two-tailed Student’s t-test using GraphPad Prism 9.3 for Windows (GraphPad Software Inc., San Diego, CA). The glucose and insulin results were analyzed by t-tests, two-way ANOVA, and repeated measures ANOVA (RMANOVA) with Bonferroni corrected pair-wise comparisons, and step-wise regression with minutes and minutes with genotype as the statistical model using the IBM-SPSS Statistics software (Chicago, IL). The metabolomics data were analyzed using two-way ANOVA with Bonferroni corrections (IBM-SPSS Statistics). In all cases, P<0.05 was considered significant.

## Results and discussion

### Expression of β-galactosidase reporter in *Ildr1*^*-/-*^ mice

**Fig 1:**
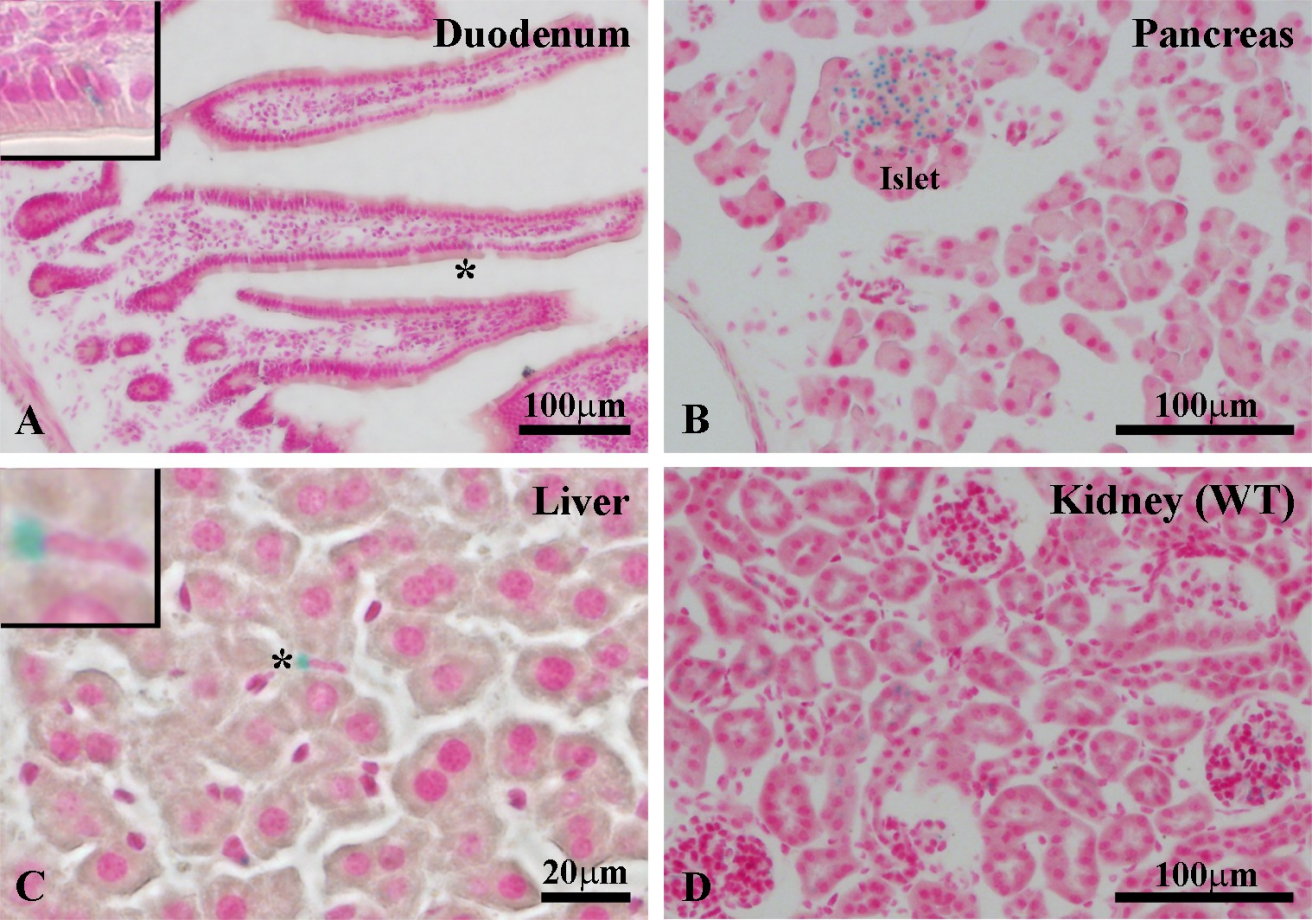
β-galactosidase reporter expression in tissues of *Ildr1*^-/-^ mice. β-galactosidase expression in (A) enteroendocrine cells of the duodenum, (B) pancreatic islets, and (C) Kupffer cells of the liver. *Inset* in panels A and C from areas marked with an asterisk in the respective panels. (D) No X-gal staining was detected in wild-type mouse kidney (control). Scale of each image is provided in the panel.

We have previously described the generation of *Ildr1*^*-/-*^ mice, in which the *Ildr1* promoter drives β-galactosidase reporter gene expression. In the duodenum, ILDR1 is selectively expressed in CCK-expressing EECs of the mucosa where it stimulates CCK secretion when exposed to a mixture of high density lipoprotein and fatty acids [2]. Fig 1 shows *Ildr1* promoter-driven β-galactosidase reporter expression (detected by X-gal staining) in three tissues. In the duodenum (Fig 1A), X-gal staining is present in isolated mucosal cells (asterisk, inset). Immunostaining with an ILDR1 specific antibody (S1 Fig) confirmed that ILDR1 is expressed on the basolateral surface of CCK-positive EECs in mouse and human intestine. In the pancreas (Fig 1B), β-galactosidase reporter expression is detected only in the islets, providing the first suggestion that ILDR1 may be involved in glucose homeostasis. No X-gal staining was observed in pancreatic acinar or ductal cells. In the liver (Fig 1C), β-galactosidase reporter expression was limited to Kupffer cells (inset), which are phagocytic macrophages that perform a scavenging function and are also involved in the initiation of immunological responses [32]. In addition, depletion of Kupffer cells leads to a reduction in cholesterol and triglycerides and the prevention of insulin resistance [33]. Thus, it appears that expression of ILDR1 in Kupffer cells is consistent with metabolic functions that could be potentially performed by ILDR1 in the liver [2]. Recently, it has been demonstrated that *Ildr1* expression is upregulated when a subset of macrophages bearing an anti-inflammatory phenotype are activated [34]. Based on these findings, *Ildr1* was categorized as an immunoregulatory gene suggesting a function for the immunoglobulin domains located on the amino terminal extracellular domain of the ILDR1 protein.

Besides these tissues, we detected X-gal staining in the Bowman capsule and tubules of the kidney (S2A Fig) [4] and in elongated spermatids of mouse testis (S2B Fig). No staining was detected in the heart (S2C Fig). In brain, X-gal staining was present in ependymal cells of the choroid plexus (S2D Fig), as well as the lateral ventricle, third ventricle, medial entorhinal cortex, presubiculum, and subiculum regions (S3 Fig). A few X-gal positive cell bodies were also visible in the hippocampus and sporadically in a few other regions of the brain that could not be clearly identified. X-gal staining was found also in mouse skin sections (S4 Fig). This discovery was fortuitous as it was observed that upon feeding a high fat diet, *Ildr1*^*-/-*^ mice had less greasy fur than wild-type littermates. In the skin, X-gal staining was present in follicular cells surrounding the hair shaft in *Ildr1*^*-/-*^ mice, whereas no staining was detected in wild-type mice. Recently, ILDR1 expression has been reported in the cochlea [6].

### Feeding behavior and metabolism in *Ildr1*^*-/-*^ mice

**Fig 2:**
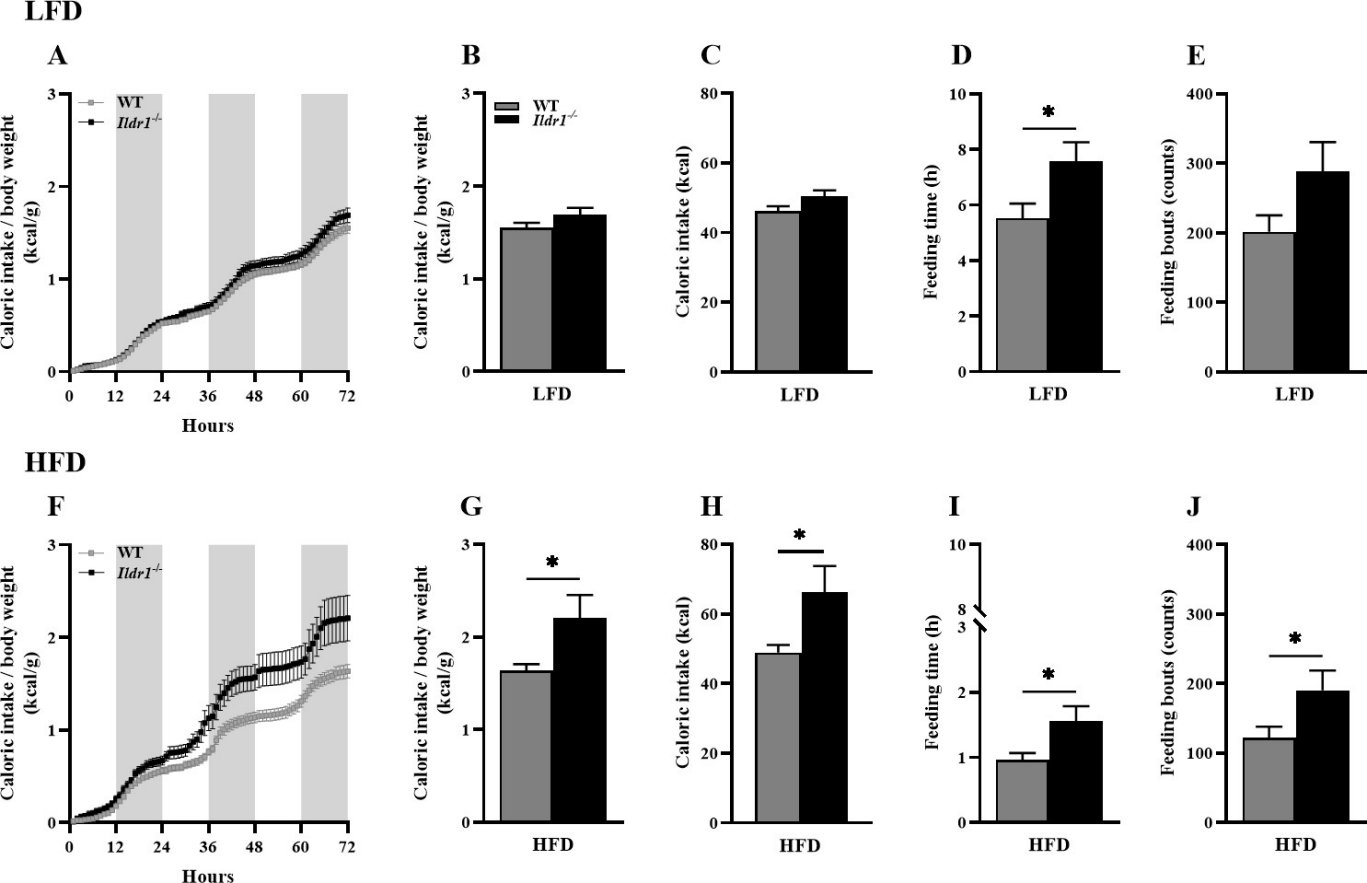
Feeding behavior of wild-type (WT) (grey line or bar) and *Ildr1*^*-/-*^ (black line or bar) mice given low fat (LFD, *top row*) or high fat diets (HFD, *bottom row*). (A) Ratio of caloric intake to body weight over 72 hr in mice fed the LFD. Light and dark cycles are represented as white or grey bars respectively over 24 hr intervals. (B) The results in panel A are expressed as the cumulative ratio for mice fed the LFD. (C) Caloric intake in mice given the LFD without normalization for body weight. (D) Cumulative time spent consuming the LFD diet [t(22) = 2.353, P = 0.028]. (E) Cumulative numbers of feeding bouts in LFD fed mice. (F) Ratio of caloric intake to body weight over 72 hr in the genotypes fed the HFD. (G) The results in panel F expressed as the cumulative ratio for mice fed the HFD [t(22) = 2.238, P = 0.036]. (H) Caloric intake in mice given the HFD without normalization for body weight [t(22) = 2.180, P = 0.040]. (I) Cumulative time spent consuming the HFD diet [t(22) = 2.417, P = 0.024]. (J) Cumulative numbers of feeding bouts in HFD groups [t(22) = 2.097, P = 0.048]. n = 12 mice/genotype/diet; *P<0.05, WT *vs*. *Ildr1*^*-/-*^.

**Fig 3:**
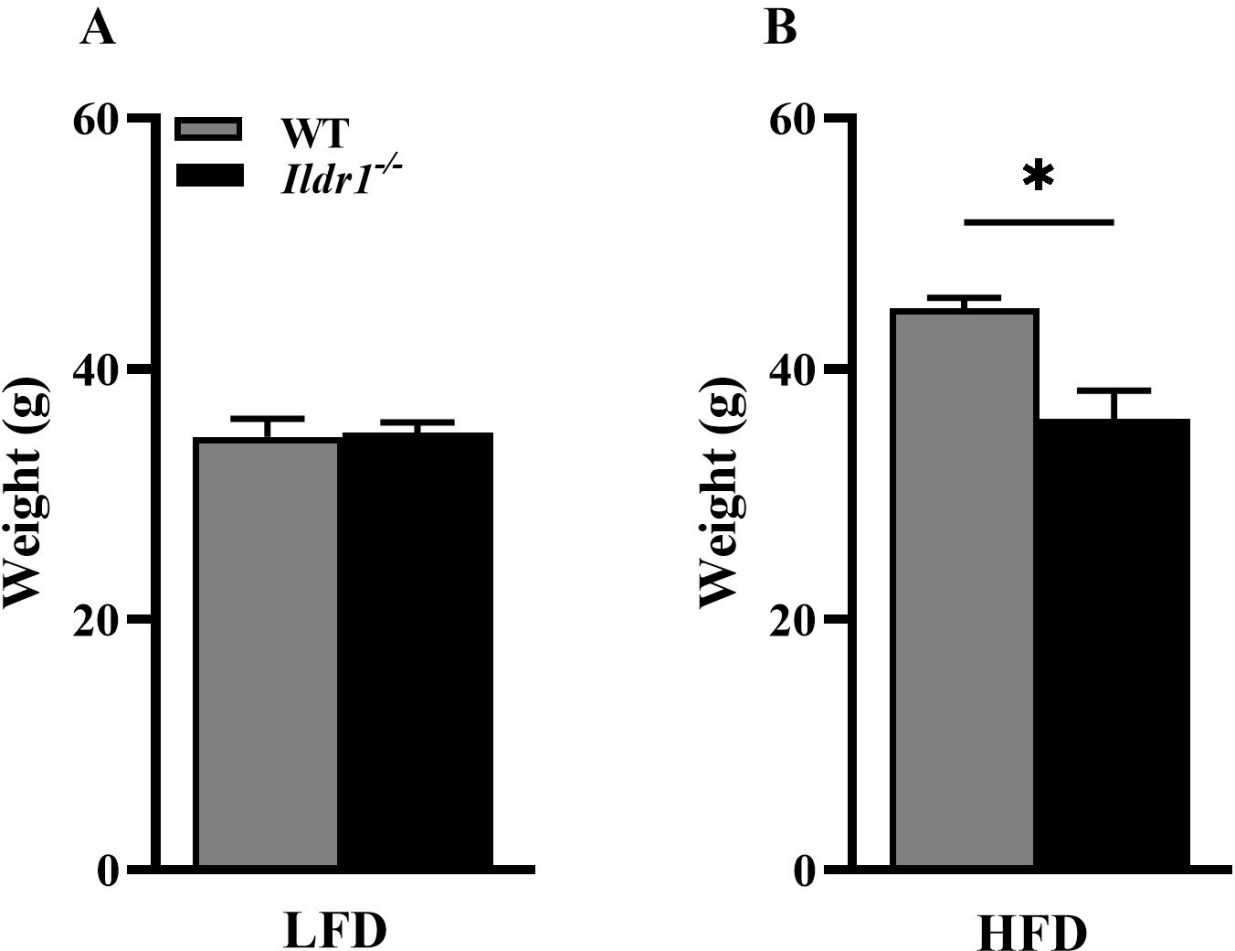
Body weights of wild-type (WT, grey bar) and *Ildr1*^*-/-*^ (black bar) mice fed a low (LFD) or high (HFD) fat diet. Mice were fed the low fat or the high fat diet for 18 days after removal from the CLAMS apparatus (total of 23 days). (A) Body weight of mice fed the LFD. (B) Body weight of mice fed the HFD [t(6) = 3.697, P = 0.010]. n = 4 mice/genotype/diet; *P<0.05, WT vs. *Ildr1*^*-/-*^.

One of the most striking features of *Ildr1*^*-/-*^ mice was their metabolic phenotype. Based on our discovery that ILDR1 is expressed in intestinal CCK cells and mediated ingested fatty acid-stimulated CCK release [2], we were interested in determining if mice lacking ILDR1 would show impaired satiety due to lack of fat-stimulated CCK secretion. To evaluate this possibility, we placed wild-type and *Ildr1*^*-/-*^ littermates in CLAMS chambers and provided Rodent 5001 chow (low fat diet, LFD) for 5 consecutive days followed by feeding a 60% fat diet (high fat diet, HFD) for 5 additional days. Fig 2 depicts the feeding behavior of pair-fed wild-type and *Ildr1*^*-/-*^ mice on low and high fat diets each over 72 hr. The caloric intakes of wild-type and *Ildr1*^*-/-*^ mice were similar on a low fat diet (Fig 2A–2C), whereas on a high fat diet the caloric intake by *Ildr1*^*-/-*^ mice was significantly enhanced (Fig 2F–2H; G, P = 0.036; H, P = 0.040). In addition, *Ildr1*^*-/-*^ mice spent more time feeding when given either the low fat (Fig 2D; P = 0.028) or high fat diet (Fig 2I; P = 0.024). Finally, the number of feeding bouts was also enhanced in *Ildr1*^*-/-*^ mice fed both diets (Fig 2E and 2J), although statistical significance was achieved only during feeding the high fat diet (P = 0.048). Thus, in accordance with an expected lack of CCK secretion and, hence, reduced satiety, *Ildr1*^*-/-*^ mice increased their consumption of the high fat diet by prolonging their feeding times and engaging in more frequent bouts of feeding. After removal from the CLAMS chambers, mice were fed either a low or high fat diet for an additional 18 days (total of 23 days). Expecting that the *Ildr1*^*-/-*^ mice would gain excessive weight, we were surprised to find that even though these mice ate more of the high fat diet, they were leaner than their wild-type littermates (Fig 3, P = 0.010). The energy content of fecal material was not evaluated in this study.

**Fig 4:**
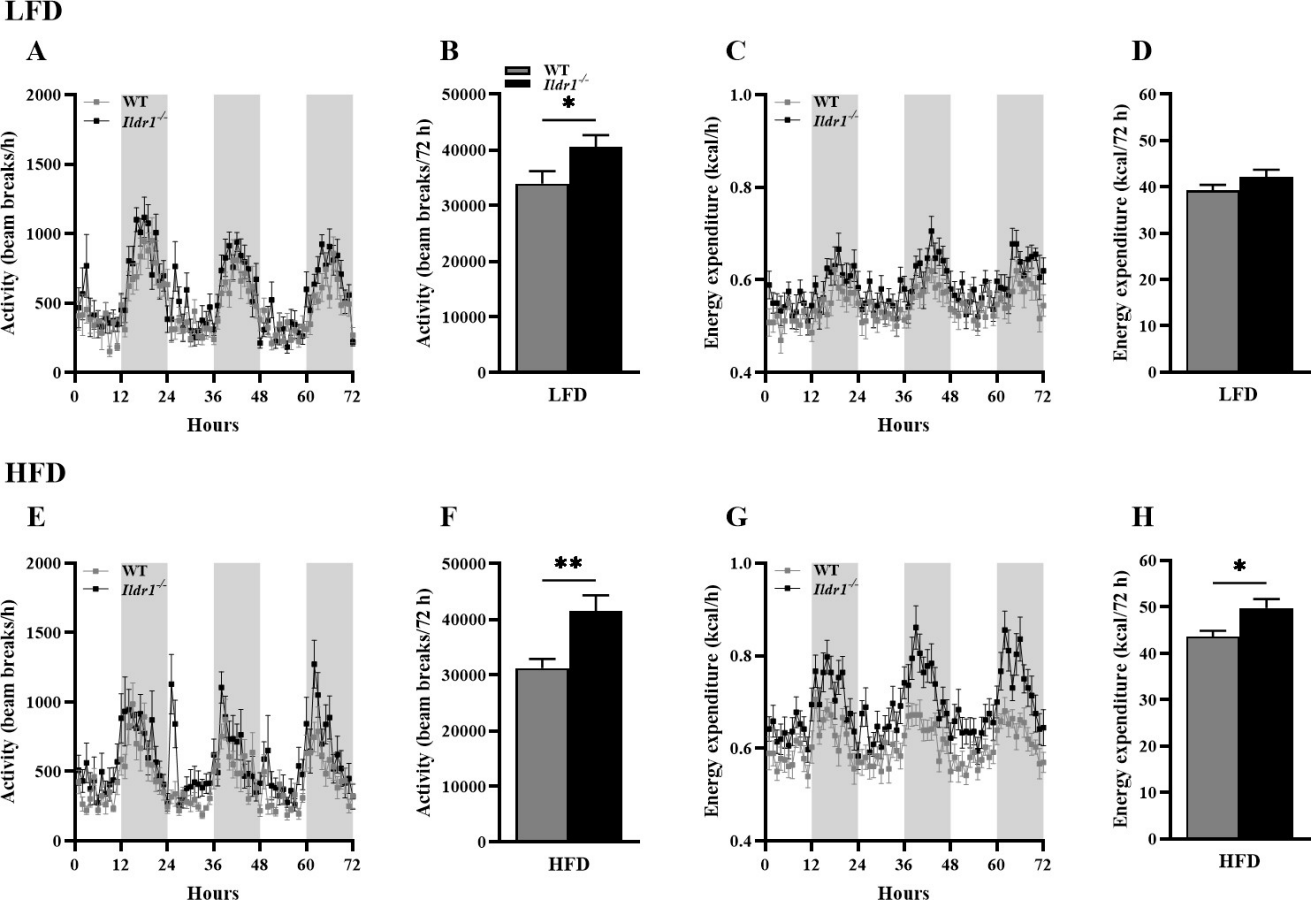
Motor activities and energy expenditures in wild-type (WT, grey lines or bars) and *Ildr1*^*-/-*^ (black lines or bars) mice when fed a low fat (LFD, *top row*) or high fat diets (HFD, *bottom row*). (A) Motor activities over 72 hr with 12-hr light and dark cycles (cycles represented as white or grey bars respectively) in wild-type and *Ildr1*^*-/-*^ mice fed the LFD. (B) Cumulative beam-breaks over 72 hr in the LFD groups [t(22) = 2.127, P = 0.045]. (C) Energy expenditure (heat production) over 72 hr with 12-hr light and dark cycles in the wild-type and *Ildr1*^*-/-*^ mice fed the LFD. (D) Cumulative energy expenditure over 72 hr in the LFD groups. (E) Motor activities over 72 hr in the wild-type and *Ildr1*^*-/-*^ mice fed the HFD. (F) Cumulative beam-breaks over 72 hr in the HFD groups [t(22) = 3.097, P = 0.005]. (G) Energy expenditure over 72 hr with 12-hr light and dark cycles in the HFD groups. (H) Cumulative energy expenditure over 72 hr in the HFD groups [t(22) = 2.744, P = 0.012]. n = 12 mice in each group; *P<0.05, **P<0.01, WT *vs*. *Ildr1*^*-/-*^.

**Fig 5:**
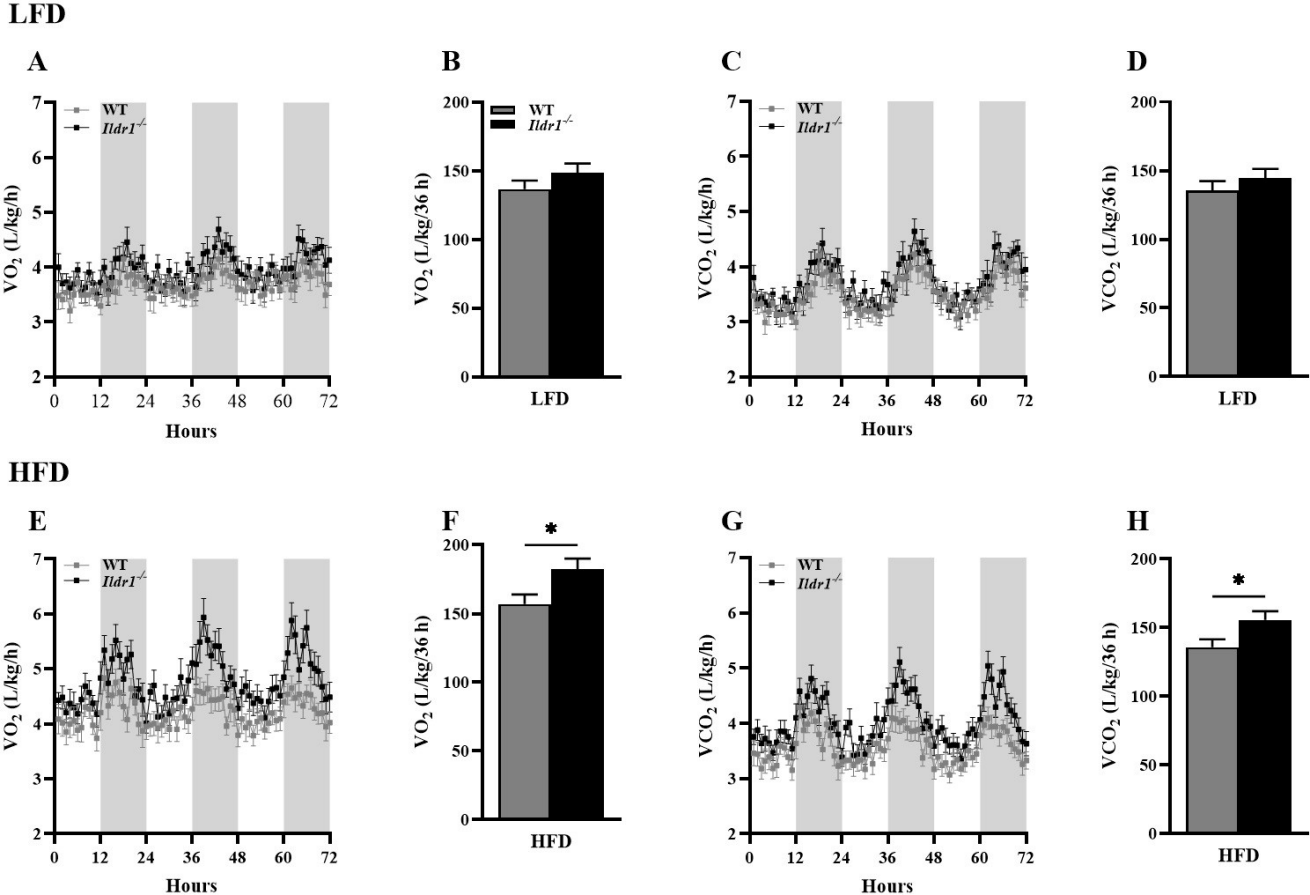
Volumes of oxygen (VO_2_) inhalation and carbon dioxide (VCO_2_) exhalation by wild-type (WT, gray) and *Ildr1*^*-/-*^ (black) mice fed either a low (LFD, *top row*) or high (HFD, *bottom row*) fat diet. (A) VO_2_ intake over 72 hr with 12-hr light and dark cycles represented as gray or black bars, respectively, in the LFD groups. (B) Cumulative VO_2_ intake during the dark cycle in the LFD groups. (C) VCO_2_ exhalation over 72 hr with 12-hr light and dark cycles in the LFD groups. (D) Cumulative VCO_2_ output during the dark cycle in the LFD groups. (E) VO_2_ intake across circadian cycles in the HFD groups. (F) Cumulative VO_2_ intake during the dark cycle in the HFD groups [t(22) = 2.402, P = 0.025]. (G) VCO_2_ exhalation over 72 hr with12 hr light and dark cycles. (H) Cumulative VCO_2_ output during the dark cycle in the HFD groups [t(22) = 2.324, P = 0.030]. n = 12 mice/genotype/diet; *P<0.05, WT *vs*. *Ildr1*^*-/-*^.

An examination of motor activity in the CLAMS apparatus revealed that the *Ildr1*^*-/-*^ mice were significantly more active irrespective of their dietary assignment than the wild-type animals (Fig 4A and 4B, P = 0.045 for LFD; Fig 4E and 4F, P = 0.005 for HFD). The increased activity was related to the increased energy expenditure in *Ildr1*^*-/-*^ mice and this expenditure was significantly higher during consumption of the high fat diet (Fig 4G–4H, P = 0.012). Since mice were housed at 73°F (22.7°C) instead of thermoneutral temperature zone for mice (28–30°C), the lower housing temperature may influence energy expenditure. Finally, *Ildr1*^*-/-*^ mice displayed increased oxygen consumption (Fig 5F, P = 0.025 for HFD) and carbon dioxide output (Fig 5H, P = 0.030 for HFD) during the dark cycles when fed a high fat diet. These results indicate that *Ildr1*^*-/-*^ mice eat more of the high fat diet than wild-type controls, and this effect may be due to reduced CCK secretion following fatty acid feeding [2]. In rats as well as in humans, administration of CCK has been reported to decrease meal frequency and inter-meal intervals, both of which appear to be compromised when feeding the high fat diet to *Ildr1*^*-/-*^ mice [35–38]. However, a new finding is that *Ildr1*^*-/-*^ mice suppress weight gain when fed a high fat diet concurrent with increased motor activity, increased energy expenditure, and higher respiration.

In addition to analyzing responses in the CLAMS apparatus, serum lipid levels were also evaluated. The cholesterol and triglyceride levels in wild-type and *Ildr1*^*-/-*^ mice were measured under fasted conditions (in mice fed the low fat diet) and non-fasting conditions (in mice fed low and high fat diets). *Ildr1*^*-/-*^ mice trended to have lower cholesterol levels under all conditions, but statistical significance was achieved only under the low fat diet, non-fasting condition (P<0.0001, S5A Fig). As anticipated, cholesterol levels in non-fasted mice fed the high fat diet were higher than animals fed the low fat diet regardless of fasting state. An examination of triglyceride levels revealed that although *Ildr1*^*-/-*^ mice trended towards lower triglyceride levels under all conditions that were tested, statistical significance between genotypes was observed only in fasted mice fed the low fat diet (P = 0.030) (S5C Fig).

### Adipose mass and adipocyte size in *Ildr1*^*-/-*^ mice

**Fig 6:**
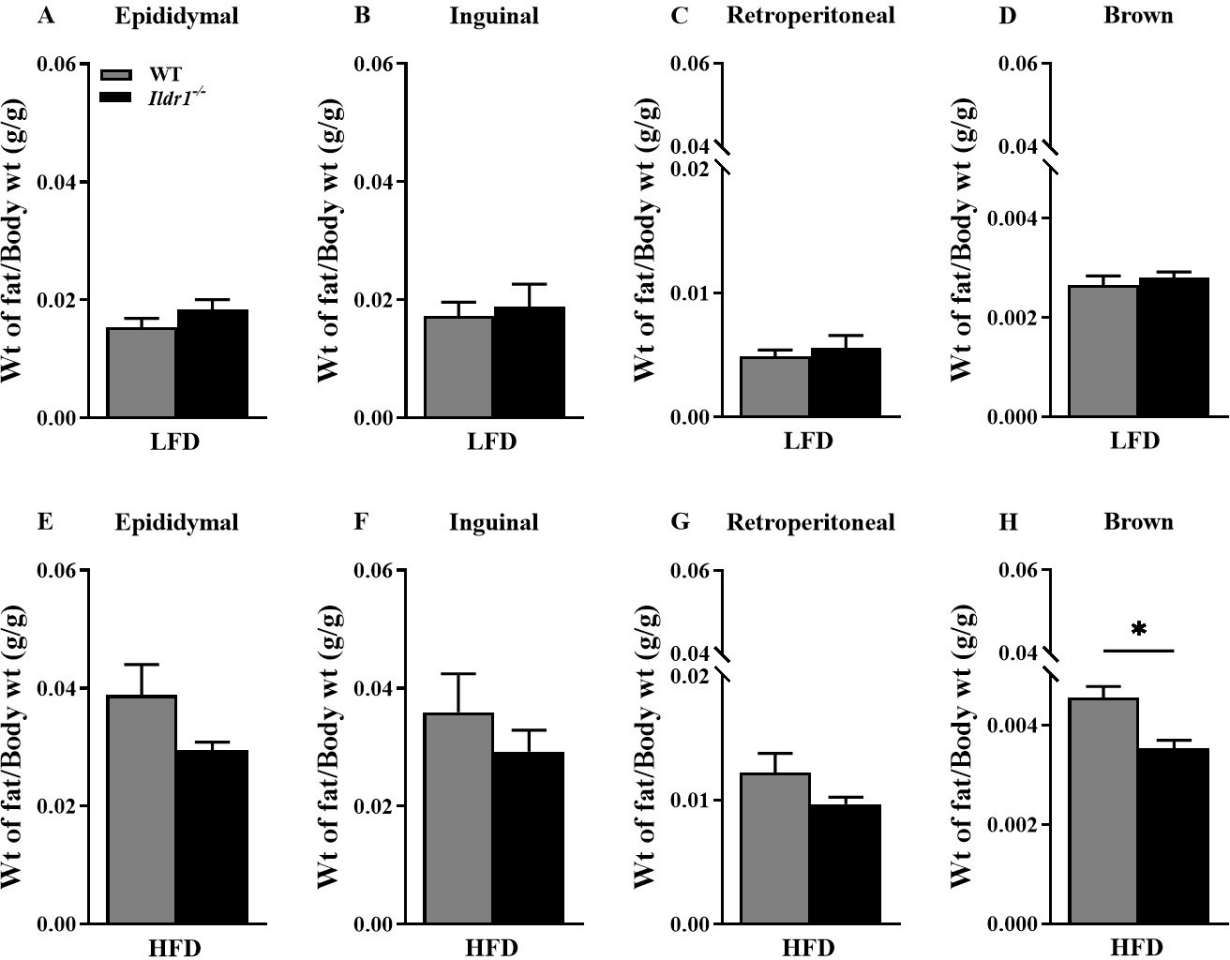
Ratios of adipose tissue to body weight in wild-type (WT, gray bars) and *Ildr1*^*-/-*^ (black bars) mice fed either a low fat (LFD) or high fat diet (HFD). Four adipose depots were collected in mice fed either a low fat (LFD) or high fat diet (HFD). (A, E) Epididymal adipose tissue weight to body weight in LFD (A) or HFD (E) fed mice. (B, F) Inguinal adipose tissue weight to body weight in LFD (B) or HFD (F) fed mice. (C, G) Retroperitoneal adipose tissue weight to body weight in LFD (C) or HFD (G) fed mice. (D, H) Brown adipose tissue weight to body weight in LFD (D) or HFD (H) fed mice; for mice fed the HFD group [t(6) = 3.590, P = 0.012]. n = 3–4 mice/genotype/diet; *P<0.05, WT *vs*. *Ildr1*^*-/-*^.

To evaluate whether the enhanced metabolic activity from the loss of ILDR1 could impact the body fat depots, four different adipose tissues were collected from wild-type and *Ildr1*^*-/-*^ mice fed a low or high fat diet. Fig 6 depicts the ratio of adipose tissue weights relative to body weight in these mice. These ratios for the white fat (epididymal, retroperitoneal, and inguinal) and brown fat depots were very similar between the genotypes when the mice were fed the low fat diet (Fig 6A–6D). While these ratios in *Ildr1*^*-/-*^ mice fed the high fat diet were decreased compared to those in wild-type mice, these values did not reach statistical significance. Nevertheless, genotype differences were found for brown fat where the ratio of its tissue weight to body weight in *Ildr1*^*-/-*^ animals fed the high fat diet was significantly lower than that in the wild-type animals (P = 0.012) (Fig 6H).

**Fig 7:**
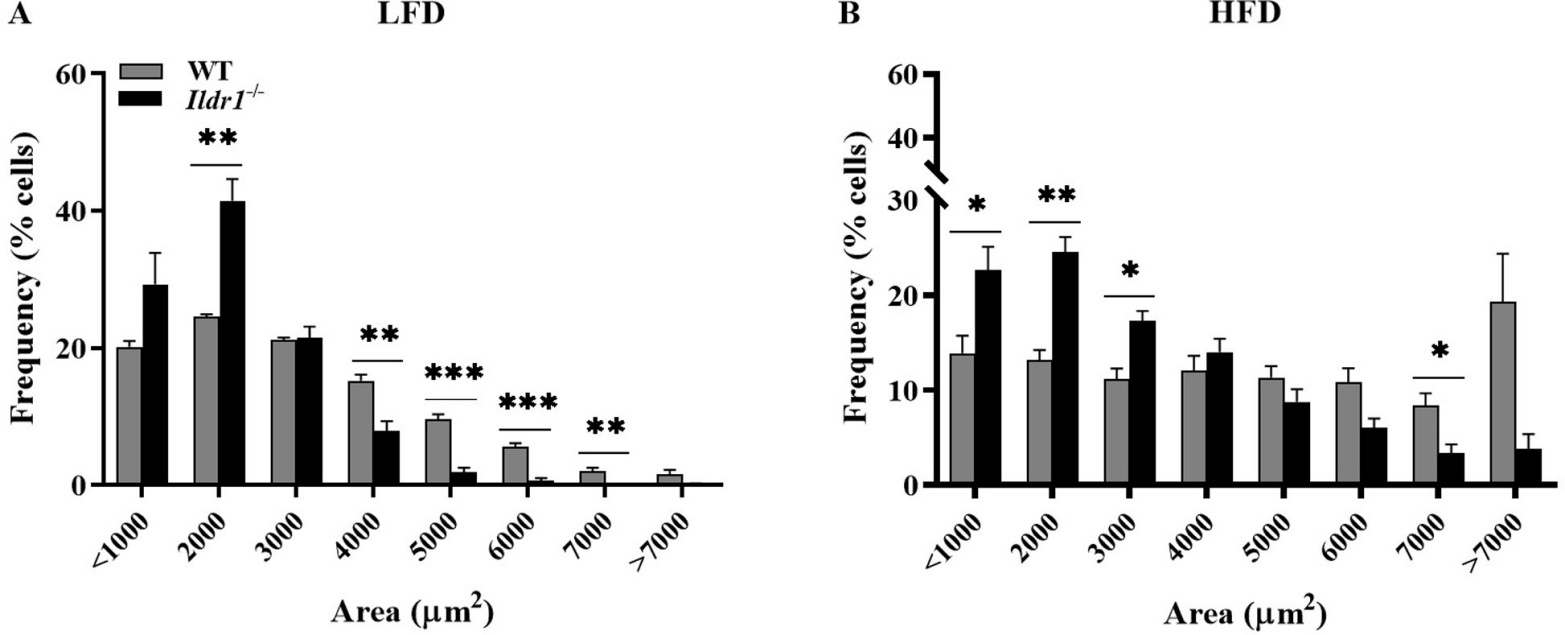
Adipocyte sizes in wild-type (WT, gray bars) and *Ildr1*^*-/-*^ mice (black bars) in a mixture of retroperitoneal and inguinal adipose tissues of mice fed either a low fat (LFD) or high fat diet (HFD). (A) LFD fed mice: 1001–2000 μm^2^ cells [t(6) = 5.373, P = 0.0017]; 3001–4000 μm^2^ cells [t(6) = 4.259, P = 0.0054], 4001–5000 μm^2^ cells [t(6) = 7.999, P = 0.0002], 5001–6000 μm^2^ cells [t(6) = 7.462, P = 0.0003], 6001–7000 μm^2^ cells [t(6) = 3.892, P = 0.0081]; (B) HFD fed mice: <1000 μm^2^ cells [t(5) = 2.929, P = 0.032], 1001–2000 μm^2^ [t(5) = 6.22, P = 0.0015], 2001–3000 μm^2^ cells [t(5) = 3.811, P = 0.012], 6001–7000 μm^2^ cells [t(5) = 2.804, P = 0.037]. n = 3–4 mice/genotype/diet; *P<0.05, **P<0.01, ***P<0.001, WT *vs*. *Ildr1*^*-/-*^.

In an additional index of body fat, we measured the size of retroperitoneal and inguinal adipocytes in wild-type and *Ildr1*^*-/-*^ mice. Irrespective of whether mice were fed the low (Fig 7A) or high (Fig 7B) fat diet, *Ildr1*^*-/-*^ mice had a higher percentage of adipocytes with smaller area, and lower percentage of adipocytes with larger area when compared with wild-type mice. However, the overall size of adipocytes increased in both wild-type and *Ildr1*^*-/-*^ mice after being exposed to high fat diet.

In order to determine whether the reductions in body fat or adipocyte cell size were associated with changes in gene expression, the mRNA of eight genes were quantitated by real-time PCR (*Adipoq*, *Lep*, *Fasn*, *Mest*, *Pparg*, *Ucp1*, *Prdm1*, and *Tnfa)*. These genes were selected based on their roles in white and/or brown adipocyte differentiation, metabolism, inflammation, or obesity. However, our results revealed no consistent trends in gene expression among the four types of fat pads between genotypes or across diet (https://research.repository.duke.edu/concern/datasets/x059c8253?locale=en&page=2).

### Plasma hormone levels in *Ildr1*^*-/-*^ mice

**Fig 8:**
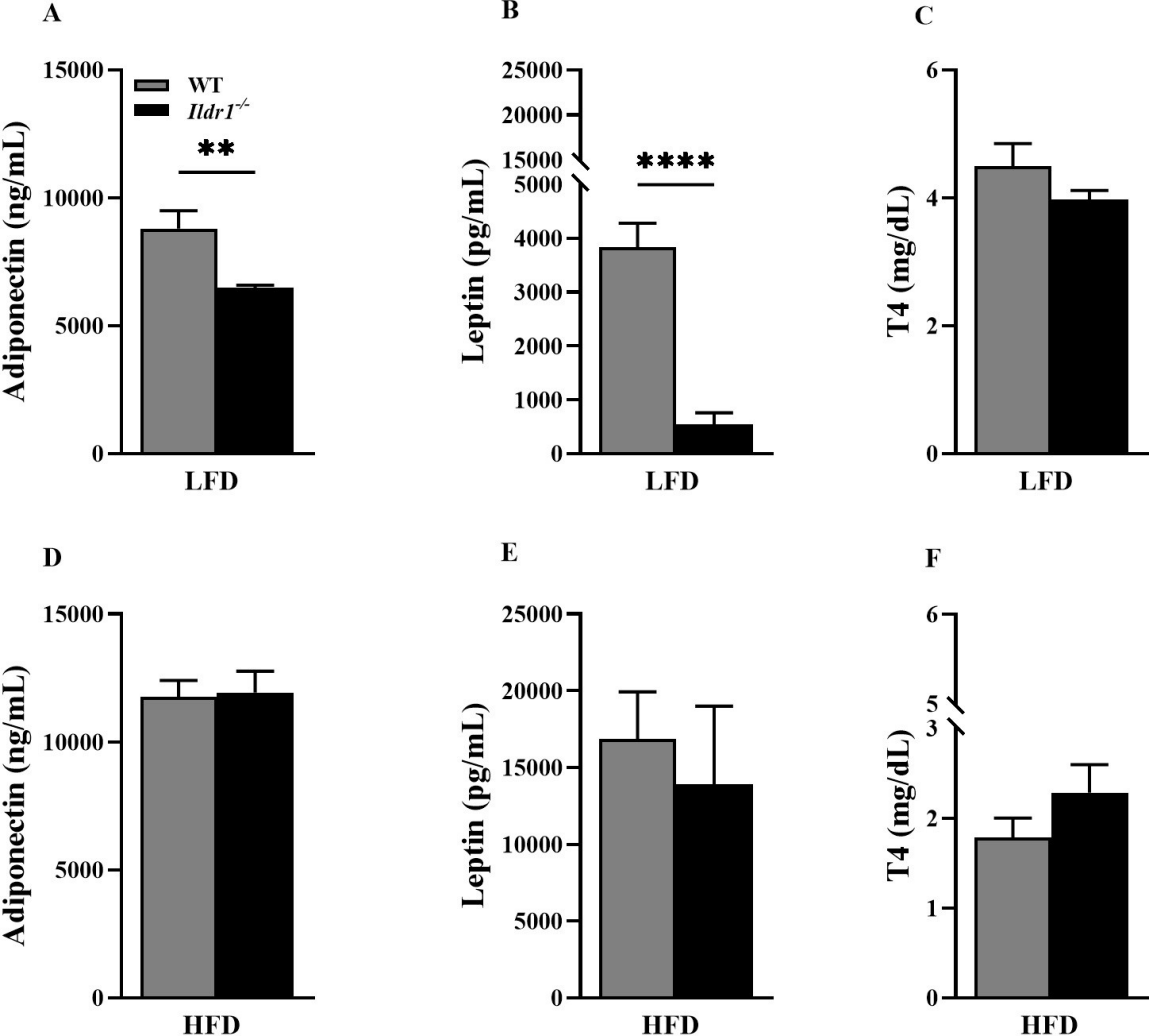
Plasma adiponectin, leptin, and T4 hormone levels in wild-type (WT, gray bars) and *Ildr1*^*-/-*^ mice (black bars) fed a low fat (LFD) or high fat (HFD) diet. (A, D) Adiponectin levels in LFD or HFD fed mice; for LFD group [t(8) = 4.130, P = 0.003]. (B, E) Leptin levels in LFD or HFD fed mice; for the LFD group [t(8) = 7.496, P<0.0001]. (C, F) T4 hormone levels in LFD or HFD fed mice. n = 4–6 mice/genotype or diet; **P<0.01, ****P<0.0001, WT *vs*. *Ildr1*^*-/-*^.

Plasma levels of three hormones, adiponectin, leptin, and free thyroxine (T4), were evaluated in wild-type and *Ildr1*^*-/-*^ mice fed either the low or high fat diet. Adiponectin is released from white fat adipocytes and increases the ability of adipocytes to store lipids [39]. Fig 8A shows that adiponectin levels were lower in *Ildr1*^*-/-*^ mice compared to wild-type mice fed a low fat diet (P = 0.003). However, under high fat diet conditions the adiponectin levels were elevated to similar extents in both genotypes (Fig 8B). It has previously been demonstrated that smaller adipocytes produce higher amounts of adiponectin [40] although a recent meta-analysis reported that at no significant correlation was observed by some researchers [41]. Our observation that adiponectin levels were higher in high fed diet fed mice (wild-type and *Ildr1*^*-/-*^), suggests that diet as well as obesity *per se* may contribute to circulating adiponectin levels. Notably, plasma adiponectin levels in mice and humans have been reported to remain unchanged [42] or to decline [43] with high fat diet feeding. In humans, adiponectin levels were shown to be directly proportional to BMI, but were also dependent on factors such as race, sex, and type of diet [44].

Like adiponectin, leptin is released from adipocytes and it inhibits food intake while concurrently stimulating energy expenditure, thereby leading to a reduction in body weight. Leptin levels decrease when energy reserves are low resulting in increased food intake. However, when energy reserves are sufficient, leptin levels increase to reduce food intake and stimulate energy expenditure [45]. Our results demonstrate that leptin levels were lower in *Ildr1*^*-/-*^ mice relative to wild-type controls fed low fat diet (Fig 8B). When mice were fed a high fat diet, leptin levels increased in both wild-type and *Ildr1*^*-/-*^ mice and these levels were visibly higher compared to mice of the same genotype that were fed low fat chow (Fig 8E). When wild-type and *Ildr1*^*-/-*^ mice were exposed to the high fat diet, leptin levels trended to be lower in *Ildr1*^*-/-*^ mice, although statistical significance was not achieved. Given the lack of decrease in fat mass in *Ildr1*^-/-^ mice fed the LFD (Fig 6), the mechanisms underlying the decline in circulating leptin remain to be investigated.

The levels of T4 were not significantly different between wild-type and *Ildr1*^*-/-*^ mice under the high fat or normal chow diet conditions (Fig 8C and 8F). Nonetheless, the levels of T4 decreased after high fat diet feeding in both genotypes. Although our experiments were conducted in mice, the literature with rats is conflicting with respect to the effect of diet on thyroid function. While, Araujo and colleagues failed to observe an effect of 60% fat diet on T4 levels [46], more recent data indicated that both T3 and T4 levels decline in rats given a high fat diet [47, 48]. This reduction in thyroid hormone levels was accompanied with structural changes in the thyroid gland, including an increase in glandular size, appearance of hypothyroid follicles, and infiltration of mast cells. Interestingly, there is some evidence to support an interaction between leptin levels and thyroid function, especially given the role of thyroxine in the maintenance of body temperature as well as regulation of uncoupling proteins [49] that can influence metabolism.

### Expression of ILDR1 in pancreatic islets

**Fig 9:**
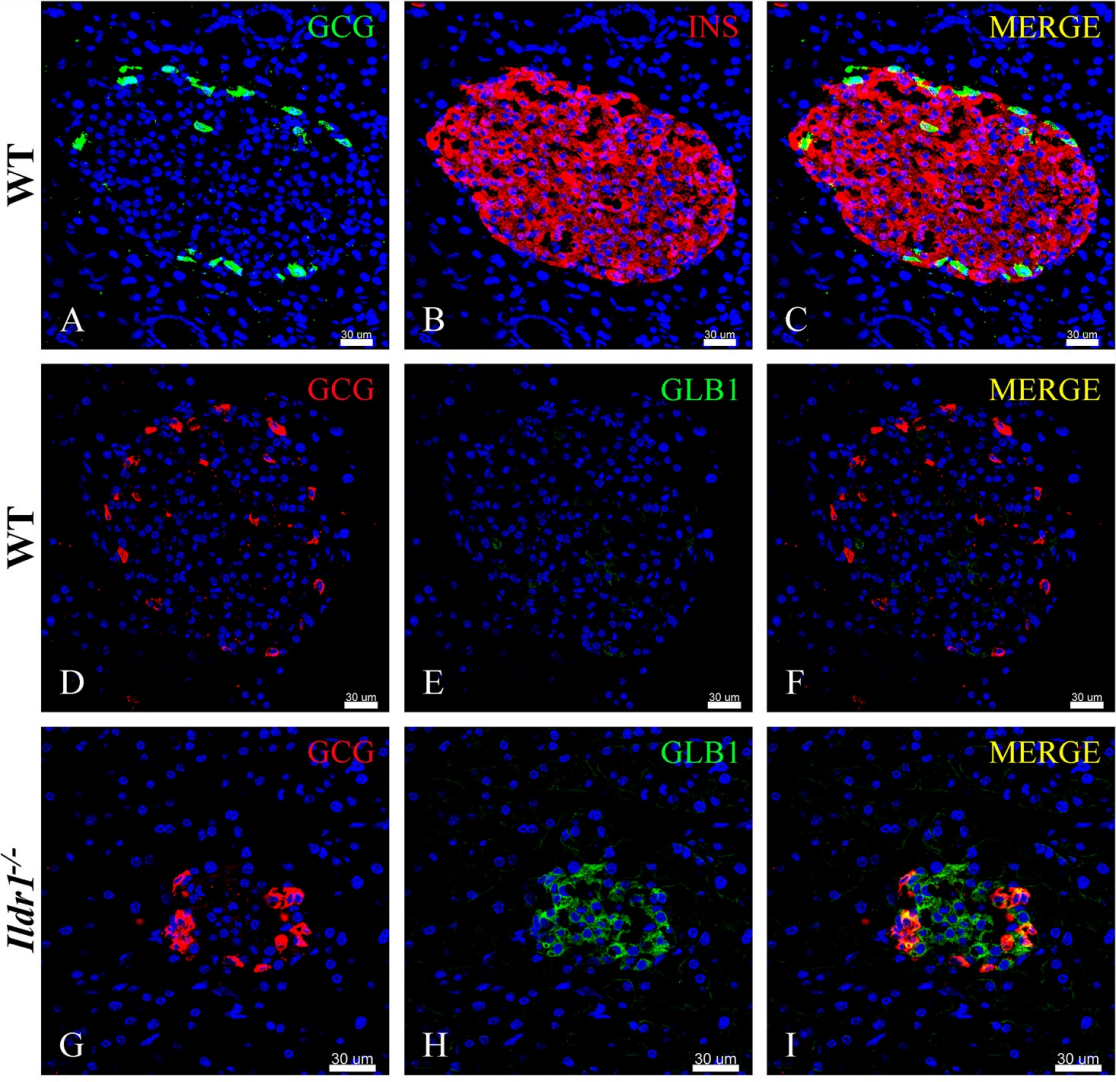
Co-localization of β-galactosidase reporter with glucagon or insulin in mouse pancreatic islets of wild-type (WT) or *Ildr1*^*-/-*^ mice. (A-C) Glucagon (green) and insulin (red) immunostaining in wild-type islets. (A) Glucagon positive alpha cells (green) are present primarily in the periphery of the islet. (B) Insulin positive beta cells (red) comprise the majority of the islet. (C) Merged image of glucagon and insulin immunostaining in a wild-type islet. (D-F) Glucagon (red) and β-galactosidase reporter (GLB1) (green) immunostaining in wild-type islet. (D) A few glucagon expressing cells (red) are present in the periphery of wild-type islets. (E) This islet is negative for β-galactosidase reporterimmunostaining. (F) Merged image of glucagon and β-galactosidase reporter immunostaining in wild-type islet. (G-I) Glucagon (red) and β-galactosidase reporter (green) immunostaining in *Ildr1*^*-/-*^ islet. (G) Similar to wild-type islet, glucagon expressing cells (red) are present in the periphery of *Ildr1*^*-/-*^ islet. (H) However, unlike wild-type islet, in *Ildr1*^*-/-*^ mice, β-galactosidase reporter (green) expression is detected. (I) Glucagon and β-galactosidase reporterantigens appear to co-localize (yellow) in the merged image. β-galactosidase reporter expression was not observed in acinar cells that surround the islets, in accordance with X-gal staining (shown in Fig 1B above). Scale bar is 30 μm in all the panels.

Our initial assessment of ILDR1 expression (Fig 1B) revealed that the β-galactosidase reporter was expressed in pancreatic islets. To further characterize the cellular localization of ILDR1 within the pancreatic islet, we immunostained wild-type and *Ildr1*^*-/-*^ pancreatic islets for glucagon (marker for alpha cells), insulin (marker for beta cells), and β-galactosidase reporter antigens. In accordance with previous reports [50], glucagon positive alpha cells were present primarily in the periphery of the wild-type murine islet (Fig 9A and 9D), whereas insulin positive beta cells comprised the majority of cells in the islet central core (Fig 9B and 9C). In wild-type islet essentially no immunostaining was detected with an antibody against β-galactosidase reporter (Fig 9E and 9F), as expected in animals lacking a transgene containing this reporter. In contrast, β-galactosidase reporter positive cells (Fig 9H) co-localized with glucagon positive alpha cells (Fig 9I) in the islets of *Ildr1*^*-/-*^ mice.

**Fig 10:**
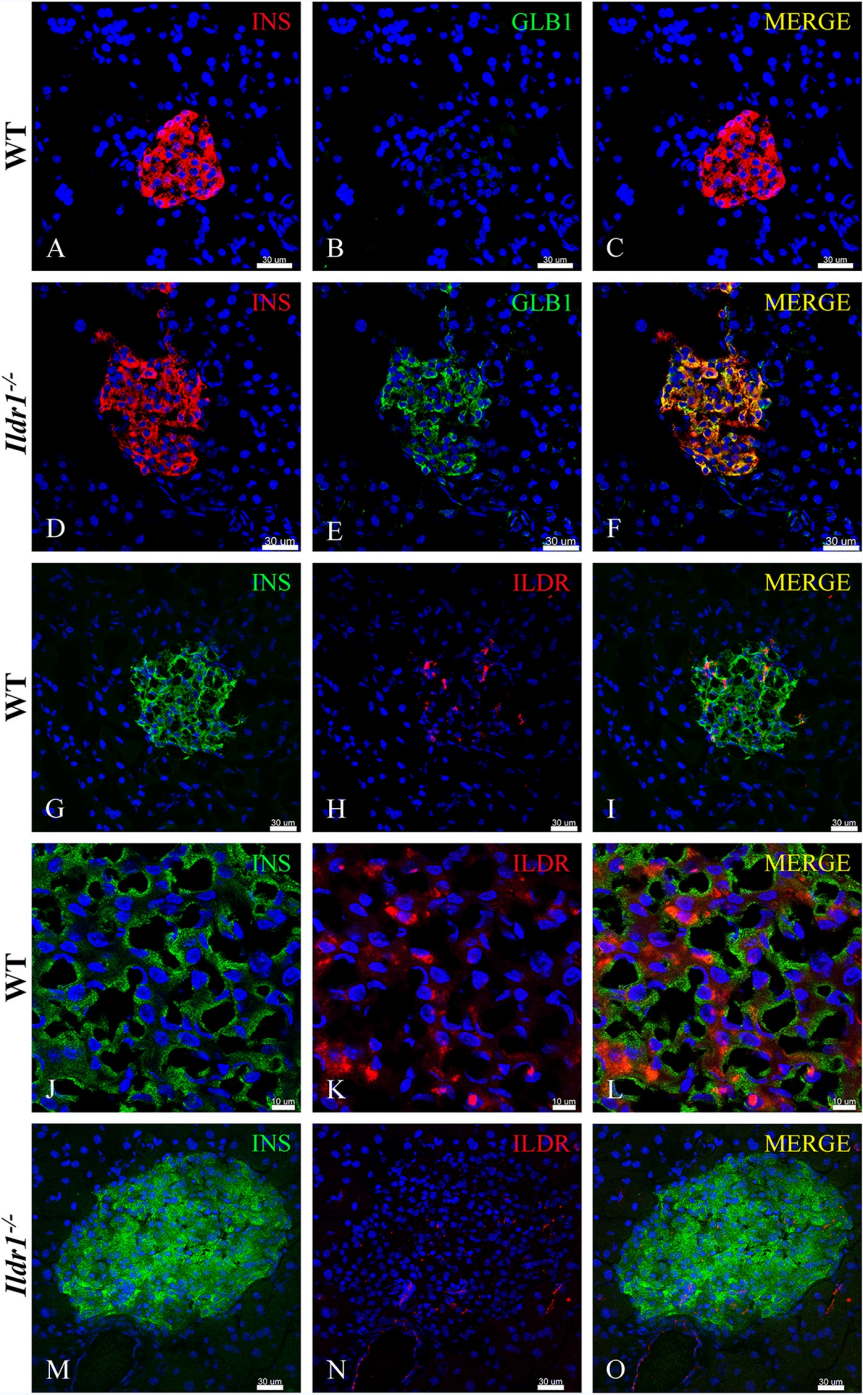
ILDR1 is expressed in pancreatic beta cells. (A-C) Insulin (red) and β-galactosidase reporter (green) staining in wild-type (WT) islets. (A) Insulin (red) expressing beta cells are present in the WT islet. (B) These cells are negative for the β-galactosidase reporter (green). (C) Merged image of insulin and β-galactosidase reporter immunostaining. (D-F) Insulin (red) and β-galactosidase reporter (green) staining in *Ildr1*^*-/-*^ islet. (D) Insulin (red) expressing beta cells are also present in the *Ildr1*^*-/-*^ islet. (E) β-galactosidase reporter (green) is expressed in *Ildr1*^*-/-*^ islet. (F) Merged image of insulin and β-galactosidase reporter immunostaining showing colocalization (yellow). (G-I) Insulin (green) and ILDR1 (red) immunostaining in wild-type (WT) islet. (G) Insulin immunostaining (green) in WT islet. (H) ILDR immunostaining (red) in WT islet. (I) While the ILDR1 immunostaining is faint, it co-localizes with insulin in islets of WT mice. (J-L) A high resolution airy scan image of insulin (green) and ILDR1 (red) in islet of WT mice. (J) Insulin immunostaining at higher magnification and resolution. (K) Corresponding ILDR1 immunostaining (green) (L) Insulin and ILDR1 appear to be spatially segregated within beta cells. (M-O) Insulin (green) and ILDR1 (red) immunostaining in *Ildr1*^*-/-*^ islet. (M) Insulin-expressing beta cells (green) are abundant in *Ildr1*^*-/-*^ islet. (N) ILDR1 immunostaining is absent in *Ildr1*^*-/-*^ islet. (O) Merged image of insulin and ILDR1 immunostaining. Scale bars are 30 μm for images showing whole islet and 10 μm for the high resolution images (panels J-L).

The co-localization of the β-galactosidase reporter with insulin in wild-type and *Ildr1*^*-/-*^ pancreatic islets is presented in Fig 10A–10F. Insulin is expressed in a majority of the cells in wild-type (Fig 10A) and *Ildr1*^*-/-*^ mice (Fig 10D) pancreatic islets. β-galactosidase reporter (green) immunostaining is not present in insulin expressing beta cells of wild-type mice (Fig 10B), but is visualized in insulin expressing cells in *Ildr1*^*-/-*^ islets (Fig 10E).

We also co-immunostained wild-type and *Ildr1*^*-/-*^ pancreas with insulin and ILDR1 antibodies (red) as presented in Fig 10G–10O. Wild-type islets have ILDR1 positive cells (Fig 10H), while staining was absent in *Ildr1*^*-/-*^ islets (Fig 10N). High resolution airy scan images (Fig 10J–10L) show that ILDR1 and insulin proteins are likely segregated within the beta cells with limited colocalization in some areas where insulin containing granules are located. Nevertheless, high intensity insulin immunostaining regions did not colocalize with high intensity of ILDR1 immunostaining, suggesting that the interaction between these two molecules is likely indirect and modulated by other signaling entities and pathways.

Two distinct roles for ILDR1 have been documented in the literature. The first is its role as a tricellular junction protein involved in the regulation of paracellular permeability in kidney tubules and, survival of cochlear hair cells [4, 6, 51]. A second function ascribed to ILDR1 is its ability to modulate lipid-stimulated CCK hormone release. However, it is not clear from our immunostaining images how ILDR1 receptor modulates islet function. The expression of the same receptor in both EECs and pancreatic islets is not physiologically unique as shown by recently published information. For example, GPR40 is expressed in EECs where it modulates incretin hormone secretion, as well as in beta cells where it participates in fatty acid-stimulated insulin secretion [52]. Similarly, GPR119 is expressed in incretin-producing L cells where it regulates glucose tolerance through dual actions in EECs and pancreatic islets [53]. Our immunostaining results demonstrate that in pancreatic islets, ILDR1 is expressed in both glucagon-producing alpha cells and insulin-producing beta cells. Glucagon levels increase during hypoglycemia to elevate blood glucose levels through actions in the liver [54]. Conversely, insulin levels increase during hyperglycemic conditions to reduce blood glucose levels. Recently, the relationship between alpha- and beta-cells has been expanded to show that paracrine signaling by alpha-cell derived proglucagon peptides help to set the cellular tone of beta-cells to control insulin secretion [55]. A parallel role for glucose-dependent insulinotropic receptor (GIPR) in alpha- to beta-cell communication has also been identified in the postprandial state [56]. GIPR is expressed on both alpha- and beta-cells of pancreatic islets, and activation of the GIPR potentiates nutrient-stimulated insulin secretion. In beta-cells, agonism of the GIPR potentiates glucose-stimulated insulin secretion [57]. In alpha-cells, GIP potentiates amino acid stimulated glucagon secretion [56], which in turn invokes alpha- to beta-cell communication to further enhance insulin release [56, 58, 59]. Whereas the expression of ILDR1 in alpha- and beta-cells suggests a possible role in alpha-to-beta-cell signaling, its role in islet function remains to be determined.

### Glucose assessments in *Ildr1*^*-/-*^ mice

**Fig 11:**
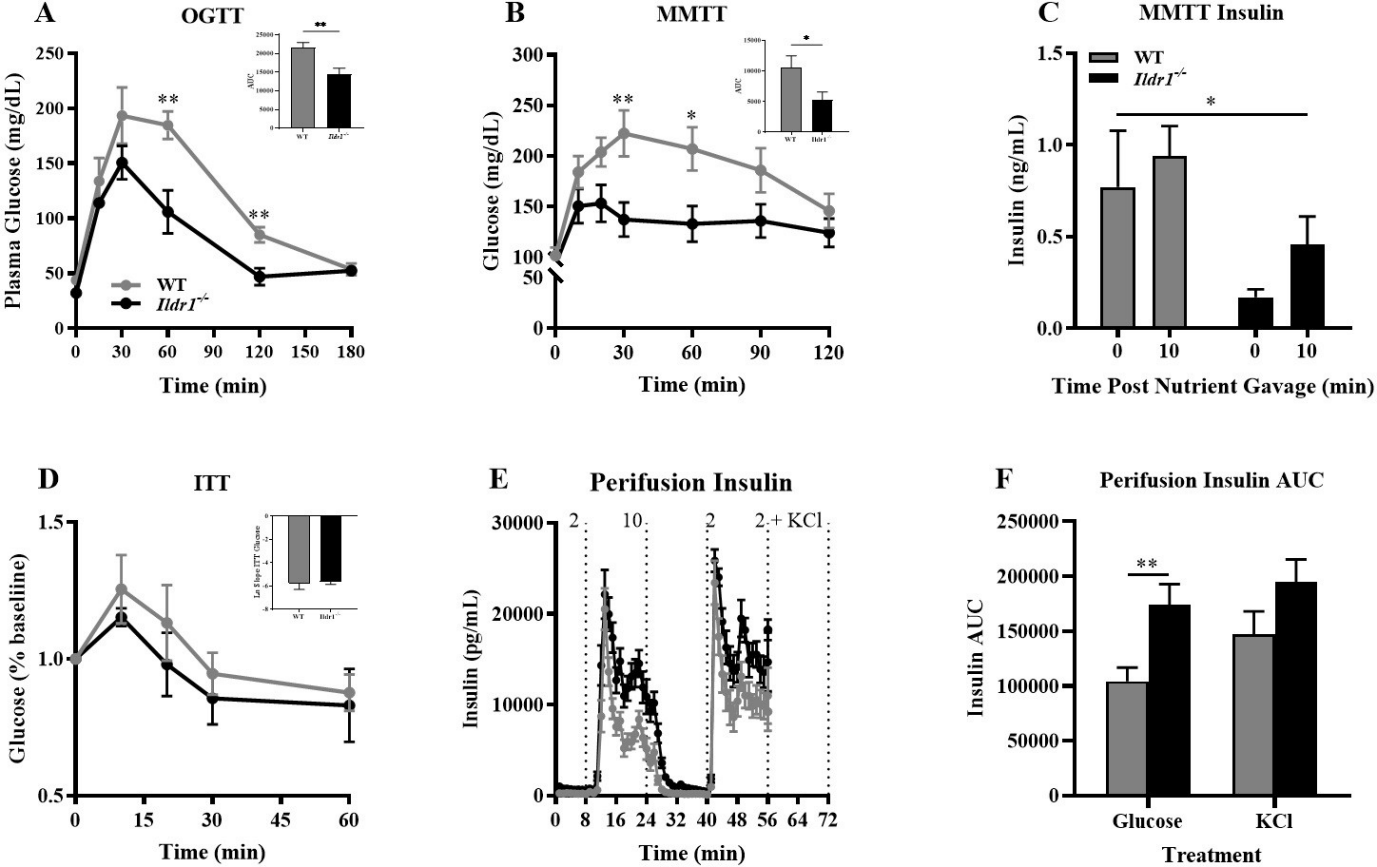
Glucose control and insulin secretion in *Ildr1*^*-/-*^ (black lines/black bars) and wild-type mice (WT, grey lines/grey bars). All tests were performed under low fat diet condition. (A) Oral glucose tolerance test (OGTT) in wild-type (WT, grey lines and grey bars) and *Ildr1*^*-/-*^ mice (black lines and black bars); RMANOVA for time [F(5,40) = 45.183, P<0.001], genotype [F(1,8) = 8.115, P = 0.022], and time by genotype [F(5,40) = 2.881, P = 0.026]. *Inset*: Area under the curve (AUC) for OGTT [t(8) = 3.541, P = 0.008]; n = 5 mice/genotype. (B) Glucose excursion in the mixed meal tolerance test (MMTT) in WT and *Ildr1*^*-/-*^ mice: RMANOVA for time [F(6,60) = 11.601, P<0.001], genotype [F(1,10) = 7.112, P = 0.024], and time by genotype [F(6,60) = 2.426, P = 0.036]; *Inset*: AUC for MMTT [t(10) = 2.335, P = 0.042]; n = 6 mice/genotype. (C) MMTT insulin measured at 0 and 10 min: step-wise regression for time [R^2^ = 0.048; F(1,23) = 1.120, P = 0.301] and genotype from baseline (time 0) to 10 min [R^2^ = 0.320; F(2,23) = 4.952, P = 0.017]. (D) Glucose excursion in the insulin tolerance test (ITT) in WT (grey lines/grey bars) and *Ildr1*^*-/-*^ mice (black lines/black bars). n = 6 mice/genotype. (E) Insulin tolerance test (ITT) monitoring insulin secretion from *ex vivo* glucose- or KCl-perfused WT and *Ildr1*^*-/-*^ islets (n = 6 mice/genotype). (F) Integrated area under the curve for glucose-stimulated insulin secretion *ex vivo* (8–24 min; from panel E) and KCl-stimulated insulin secretion (40–56 min); for glucose [t(22) = 3.082, P = 0.005, two-tailed]. *P<0.05, **P<0.01 WT *vs*. *Ildr1*^*-/-*^.

In order to assess the effect of *Ildr1* deletion on glucose homeostasis, we performed a series of tests to measure glucose tolerance, insulin sensitivity, and insulin secretion (Fig 11). *Ildr1*^*-/-*^ mice demonstrated enhanced glucose tolerance compared to wild-type mice when administered an oral glucose challenge (AUC P-value = 0.008) (Fig 11A). To invoke the full incretin effect and recruit both alpha- and beta-cells [56], we also conducted a mixed meal tolerance test (Fig 11B and 11C). In the MMTT, *Ildr1*^*-/-*^ mice had lower glycemia compared to wild-type mice (AUC P-value = 0.042) (Fig 11B) and this reduction in glycemia was accompanied with a larger increment in insulin secretion response to the meal in the *Ildr1*^*-/-*^ relative to wild-type mice, albeit from a lower baseline value (P = 0.017) (Fig 11C). The lower basal plasma insulin levels in *Ildr1*^*-/-*^ mice suggested these mice have improved insulin sensitivity relative to the wild-type controls. However, an insulin tolerance test (ITT, Fig 11D) failed to reveal significant differences between the genotypes, suggesting that insulin secretion, but not insulin sensitivity, is the primary driver in the two glucose tolerance tests. We tested this hypothesis in an *ex vivo* islet perifusion system (Fig 11E and 11F). In response to high glucose (10 mM), perifused islets from *Ildr1*^*-/-*^ mice showed a significantly more robust response that islets from wild-type mice (P = 0.022) (Fig 11F). When islets were depolarized with 30 mM KCl, both genotypes responded similarly (Fig 11F) suggesting an improvement in the mechanisms that facilitate substrate-driven insulin secretion. The improved glycemia in *Ildrl*^-/-^ mice is presumably driven by the enhanced responsivity of the islet to glucose (Fig 11A and 11E). Whether there is an enhanced response to other insulin secretagogues like amino acids or incretin peptides is currently unknown. Further studies of the role(s) of ILDR1 in regulation of beta-cell metabolism and function are warranted.

### Metabolomics profiles in *Ildr1*^*-/-*^ mice

Due to the effects of *Ildr1*^*-/-*^ gene deletion eliciting changes in multiple variables involved in metabolic homeostasis (i.e., food intake, energy expenditure, insulin secretion, and glucose tolerance), we used targeted metabolomics to investigate changes in plasma and tissue metabolites produced by genetic deletion of this receptor. Our analysis included a set of 15 amino acids, 65 acylcarnitine species, 7 organic acids, including multiple TCA cycle intermediates, lactate and pyruvate, 67 acyl CoA species, and 21 ceramides.

Overall, distinct differences in dietary responses between *Ildr1*^*-/-*^ mice compared to wild-type mice were not reflected in large changes in the metabolite profiles in the tissues surveyed (epididymal fat, blood, liver, and gastrocnemius muscle; S6 Fig).

**Fig 12:**
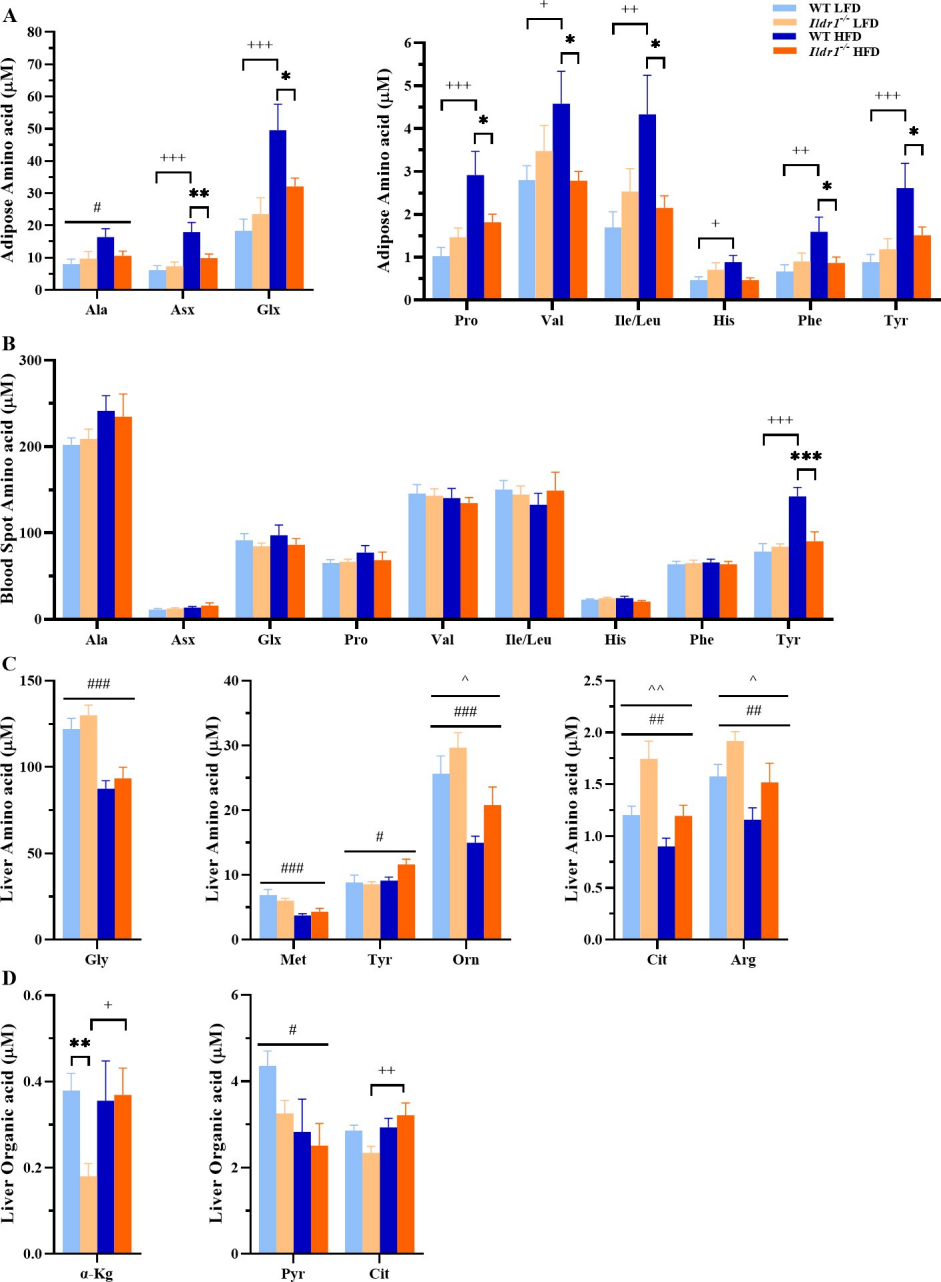
Metabolite profiles of wild-type (WT) and *Idlr1*^*-/-*^ mice fed a low fat diet (LFD) and high fat diet (A) Adipose amino acid profile for Ala: diet [F(1,22) = 4.436, P = 0.047]; for Asx: diet [F(1,22) = 14.686, P<0.001], genotype by diet [F(1,22) = 6.057, P = 0.022]; and for Glx: diet [F(1,22) = 13.319, P = 0.001], genotype by diet [F(1,22) = 4.295, P = 0.050]; for Pro: diet [F(1,22) = 12.128, P = 0.002], genotype by diet [F(1,22) = 5.753, P = 0.025]; for Val: genotype by diet [F(1,22) = 4.971, P = 0.036]; Ile/Leu: genotype by diet [F(1,22) = 6.604, P = 0.017]; for His: genotype by diet [F(1,22) = 5.645, P = 0.027]; Phe: genotype by diet [F(1,22) = 4.495, P = 0.045]; and for Tyr: diet [F(1,22) = 9.309, P = 0.006], genotype by diet [F(1,22) = 4.351, P = 0.049]. (B) Blood spot amino acid profile for Tyr: genotype [F(1,23) = 7.552, P = 0.011], diet [F(1,23) = 17.184, P<0.001], genotype by diet [F(1,23) = 11.497, P = 0.003]. (C) Liver amino acid profile for Gly: diet [F(1,23) = 35.189, P<0.001]; for Met: diet [F(1,23) = 16.715, P<0.001]; for Tyr: diet [F(1,23) = 4.528, P = 0.044]; for Orn: genotype [F(1,23) = 4.192, P = 0.052], diet [F(1,23) = 16.240, P<0.001]; for Cit: genotype [F(1,23) = 10.913, P = 0.003], diet [F(1,23) = 11.373, P = 0.003]; and for Arg: genotype [F(1,23) = 7.633, P = 0.011], diet [F(1,23) = 10.412, P = 0.004]. (D) Liver organic acid profile for αKg: genotype by diet [F(1,21) = 4.876, P = 0.038]; for Pyr: diet [F(1,23) = 5.571, P = 0.027], and for Cit: diet [F(1,23) = 5.857, P = 0.024], genotype by diet [F(1,23) = 4.227, P = 0.051]. n = 5–8 samples/group. *P<0.05, **P<0.01, ***P<0.001, WT *vs*. *Ildr1*^*-/-*^ within diet; +P<0.05, ++P<0.01, +++P<0.001, low *vs*. high fat diet within genotype; ^P<0.05, ^^P<0.01, overall genotype effect; #P<0.05, ##0.01, ###P<0.001, overall diet effect.

Both *Ildr1*^*-/-*^ and wild-type mice exhibited significant changes in the levels of multiple metabolites when fed high-fat *versus* low fat diet, particularly in liver where acylcarnitine, acyl-CoA’s and certain amino acids displayed robust diet-dependent changes (Fig 12). However, most of these metabolites were similarly affected by diet across the tissues in the two genotypes (S6 Fig). A prominent exception was the amino acid profile in adipose tissue, where the levels of nine amino acids increased in response to high fat diet in wild-type, but not *Ildr1*^*-/-*^ mice (Fig 12A). Interestingly, of these nine amino acids, only tyrosine showed the same pattern of response in blood spots, with an increase in response to the high fat diet in the wild-type, but not in the *Ildr1*^*-/-*^ mice (Fig 12B). In contrast, among six amino acids that changed significantly in liver in response to diet in the *Ildr1*^*-/-*^, five of these also changed in response to diet in wild-type mice (Fig 12C). Surprisingly, in contrast to adipose and blood spots, hepatic tyrosine levels were influenced by high fat feeding in the *Ildr1*^*-/-*^ mice, but not in the wild-type controls. Finally, despite these few instances of differential responses to diets in the two genotypes, comparisons of metabolite levels across genotypes revealed very few significant differences, irrespective of dietary condition. One exception was a change in the liver organic acids α-ketoglutarate and citrate in *Ildr1*^*-/-*^ relative to wild-type mice fed only the low fat diet (Fig 12D). Overall, we found no evidence of dramatic changes in metabolic fuel selection or activities of key metabolic pathways, with the possible exception of a broad effect on amino acid balance in adipose tissue.

## Conclusion

Our results show that ILDR1 is expressed in several tissues where it may serve diverse physiologic roles. *Ildr1*^*-/-*^ mice have a modified metabolic phenotype compared to wild-type controls in which they consume more of a high fat diet, but do not gain adipose mass to the same degree, presumably due to higher metabolism and locomotor activity. In addition, ILDR1 is expressed in alpha and beta cells of the pancreas, where it appears to play a role in insulin secretion through an unknown mechanism. Further investigations are needed to elucidate the functional role that ILDR1 may play in multiple organs throughout the body.

## Supporting information

S1 Fig ILDR1 immunostaining reveals basolateral expression in CCK cells of mouse and human duodenum. (A-C) In the mouse duodenum, CCK immunopositive cells (green) express ILDR1 (red) protein. An overlay of the two channels is shown in panel C. (D-F) In the human duodenum, ILDR1 (red) immunostaining is present in CCK-expressing cells (green). ILDR1 immunostaining was located in the basolateral region of the CCK positive enteroendocrine cells. Scale bars are 20 μm for mouse and 10 μm for human images.
(TIF)

S2 Fig β-galactosidase reporter expression in tissues of *Ildr1*^-/-^ mice. β-galactosidase reporter expression in (A) kidney, (B) testis, (C) heart, and (D) brain choroid plexus. No X-gal staining was detected in heart (C). Scale bar is shown in the bottom right of each panel.
(TIF)

S3 Fig X-gal staining in transverse section of *Ildr1*^*-/-*^ brain. (A) Bilateral X-gal staining (blue) is present in the medial entorhinal cortex, presubiculum, and subiculum regions. (B) Higher magnification of the subiculum region showing staining in ependymal cells of the lateral ventricle (arrow). Scale bar: Panel A = 500 μm; Panel B = 100 μm. Abbreviations: Bic: brachium of inferior colliculus, CA1: CA1 field of hippocampus; D3V: dorsal 3^rd^ ventricle; hf: hippocampal sulcus; mEnt: medial entorhinal cortex; LV: lateral ventricle; S: subiculum.
(TIF)

S4 Fig ILDR1 is expressed in hair follicles. (A) Wild-type (WT) mice fed a high fat diet have greasy fur. (C) *Ildr1*^-/-^ mouse fur is unaffected by the high fat diet. (B) Wild type mice show no X-gal staining in the skin. (D) X-gal staining is present in the hair follicle and follicular cells (*) surrounding the hair shaft in *Ildr1*^-/-^ mice. The nuclei are counterstained with nuclear fast red. Scale bar is 20 μm and scale is the same for panels B and D.
(TIF)

S5 Fig Serum cholesterol and triglyceride levels in wild-type (WT, gray bars) or *Ildr1*^*-/-*^ mice (black bars) fed low fat (LFD) or high fat (HFD) diets. (A) Cholesterol in LFD fed mice was measured in fasting and non-fasting conditions; non-fasting [t(6) = 11.74, P<0.0001]. (B) Cholesterol levels in HFD fed WT and *Ildr1*^*-/-*^ mice did not achieve statistical significance. (C) Triglycerides in LFD fed mice was measured in fasting [t(6) = 2.833, P = 0.0298] or non-fasting conditions. (D) Triglyceride levels in HFD fed WT and *Ildr1*^*-/-*^ mice did not achieve statistical significance. n = 4 mice/genotype/treatment; *P<0.05, ***P<0.0001, WT vs. *Ildr1*^*-/-*^.
(TIF)

S6 Fig Heat maps of low fat diet (LFD) and high fat diet (HFD) fed wild-type (WT) and *Idlr1*^*-/-*^ mice. (A) Heat map displaying effects of diet and genotype on the metabolite profiles in adipose tissue. (B) Heat maps displaying effects of diet and genotype on the metabolite profiles in blood. (C) Heat map displaying effects of diet and genotype on the metabolite profiles in liver. (D) Heat map displaying effects of diet and genotype on the metabolite profiles in gastrocnemius muscle.
(TIF)

## Abbreviations

ILDR1: Immunoglobulin-like domain containing receptor 1
CCK: cholecystokinin
EEC: enteroendocrine cell
CLAMS: Comprehensive Lab Animal Monitoring System
X-gal: 5-bromo-4-chloro-3-indolyl-β-D-galactopyranoside
OGTT: oral glucose tolerance test
MMTT: mixed meal tolerance test
ITT: insulin tolerance test
