# Supplementary figures and images for: Ildr1 gene deletion protects against diet-induced obesity and hyperglycemia
Source: PLoS One. 2022 Jun 24;17(6):e0270329. doi: 10.1371/journal.pone.0270329 (PMC9231709; doi:10.1371/journal.pone.0270329)

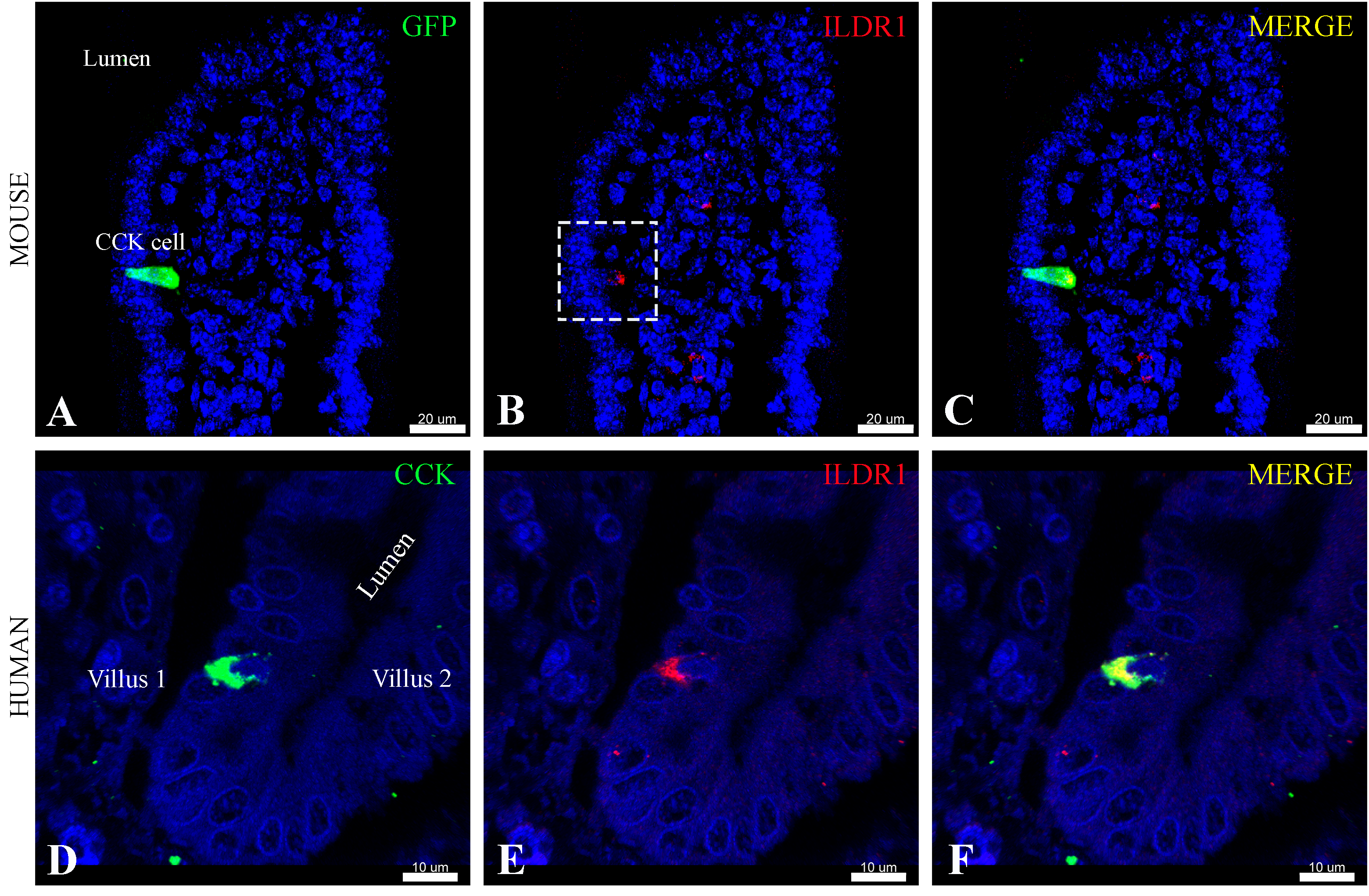

Supplement: S1 Fig — (A-C) In the mouse duodenum, CCK immunopositive cells (green) express ILDR1 (red) protein. An overlay of the two channels is shown in panel C. (D-F) In the human duodenum, ILDR1 (red) immunostaining is present in CCK-expressing cells (green). ILDR1 immunostaining was located in the basolateral region of the CCK positive enteroendocrine cells. Scale bars are 20 μm for mouse and 10 μm for human images. (TIF) [file pone.0270329.s001.tif]

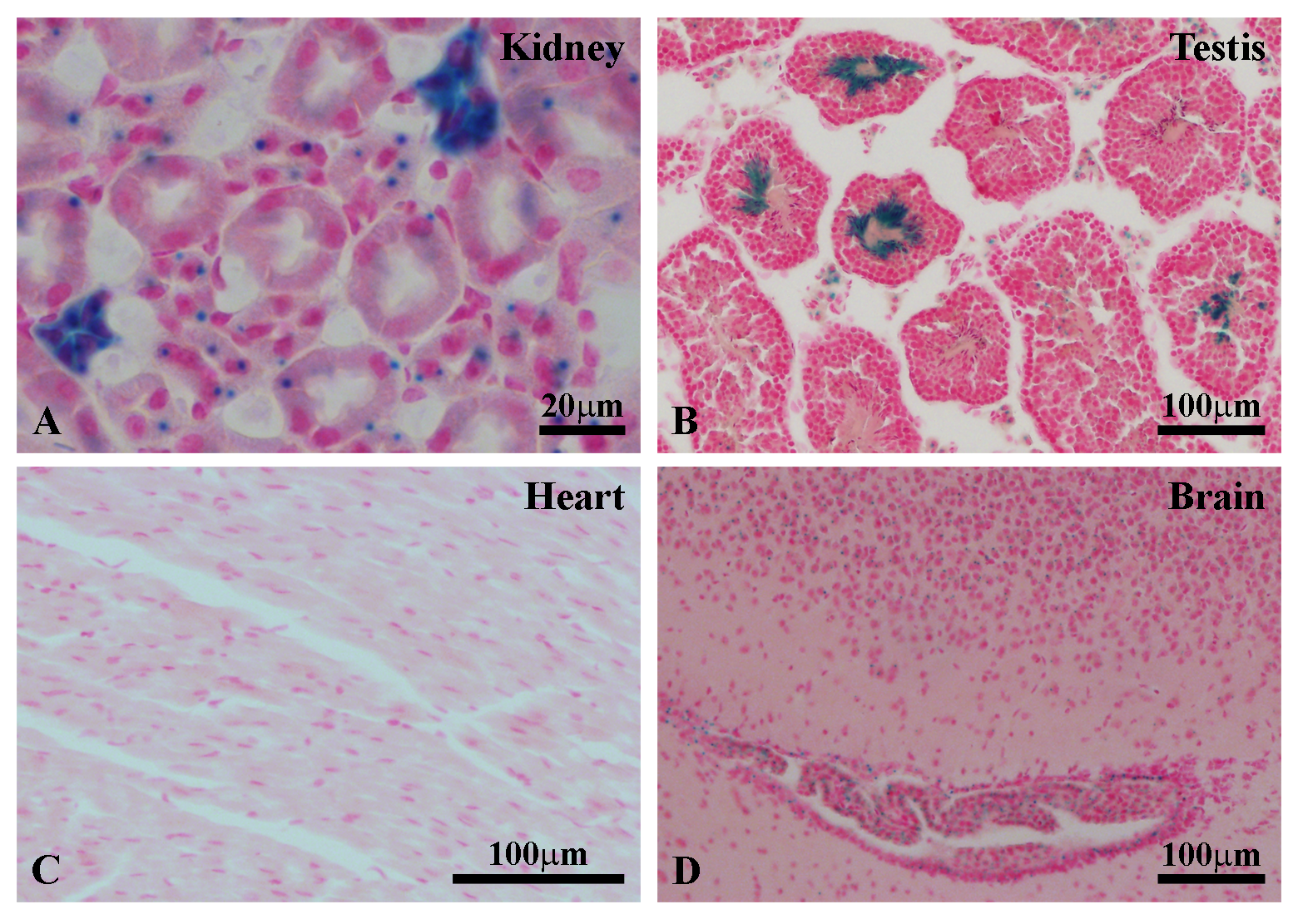

Supplement: S2 Fig — β-galactosidase reporter expression in (A) kidney, (B) testis, (C) heart, and (D) brain choroid plexus. No X-gal staining was detected in heart (C). Scale bar is shown in the bottom right of each panel. (TIF) [file pone.0270329.s002.tif]

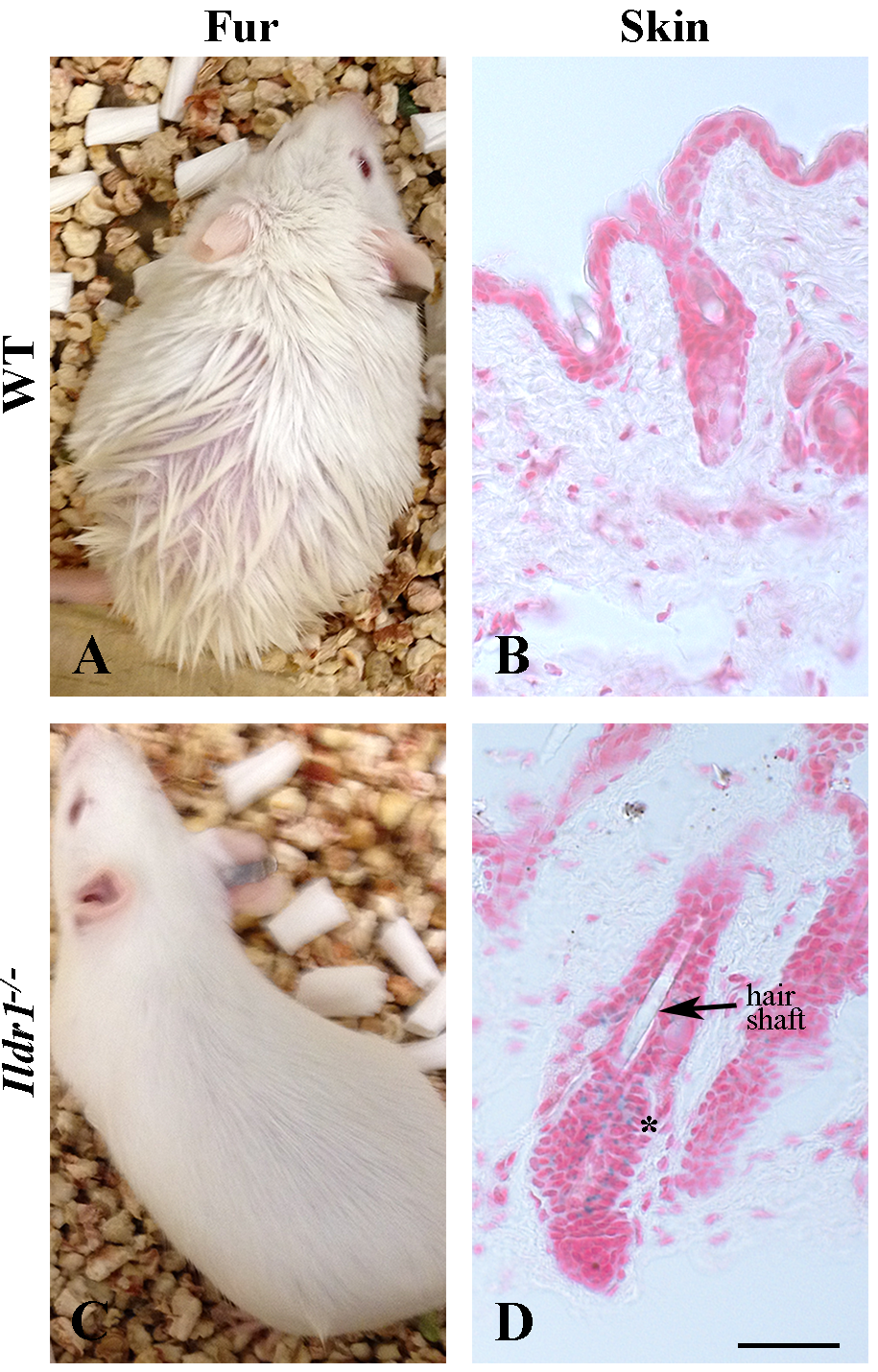

Supplement: S3 Fig — (A) Bilateral X-gal staining (blue) is present in the medial entorhinal cortex, presubiculum, and subiculum regions. (B) Higher magnification of the subiculum region showing staining in ependymal cells of the lateral ventricle (arrow). Scale bar: Panel A = 500 μm; Panel B = 100 μm. Abbreviations: Bic: brachium of inferior colliculus, CA1: CA1 field of hippocampus; D3V: dorsal 3rd ventricle; hf: hippocampal sulcus; mEnt: medial entorhinal cortex; LV: lateral ventricle; S: subiculum. (TIF) [file pone.0270329.s003.tif]

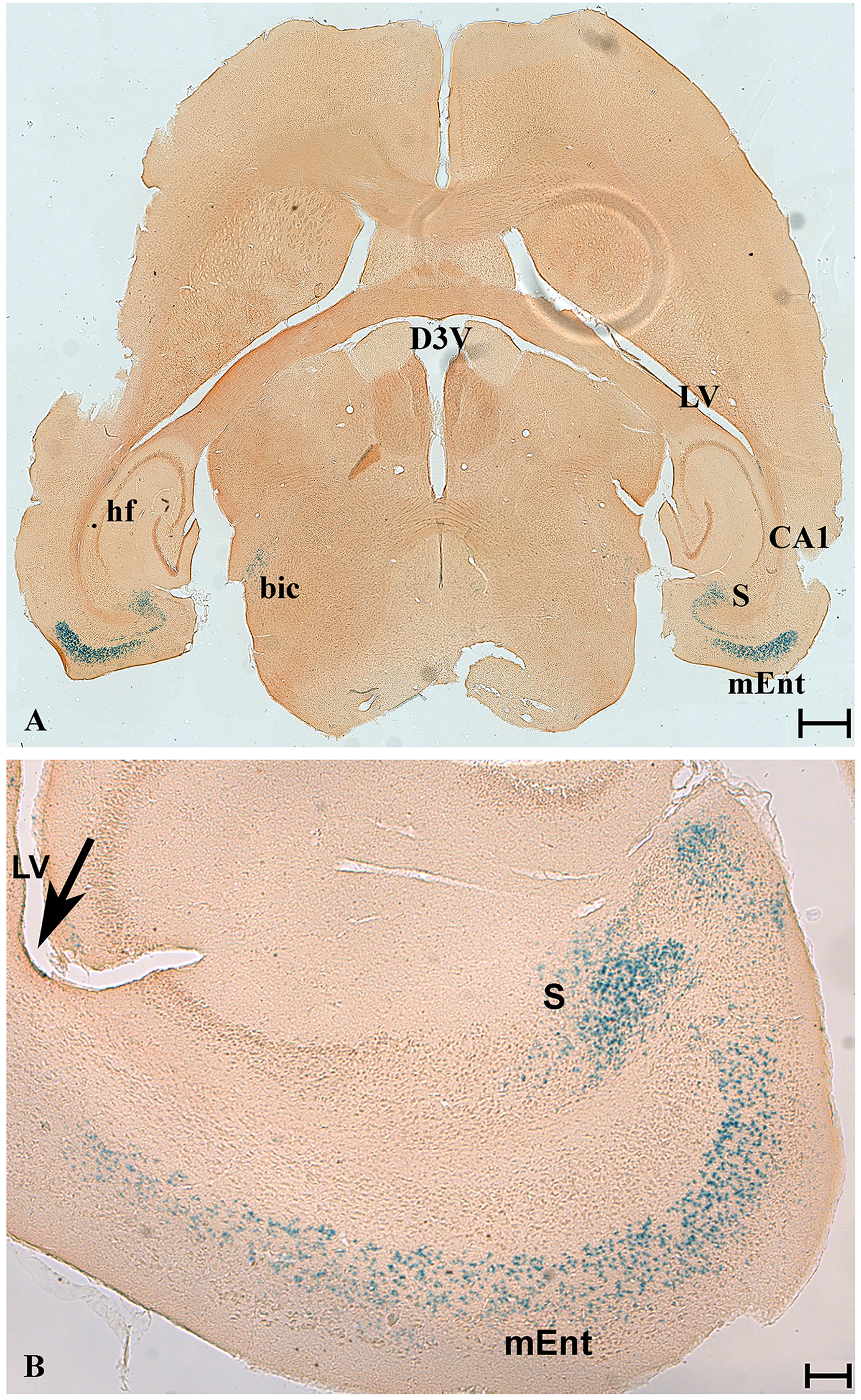

Supplement: S4 Fig — (A) Wild-type (WT) mice fed a high fat diet have greasy fur. (C) Ildr1-/- mouse fur is unaffected by the high fat diet. (B) Wild type mice show no X-gal staining in the skin. (D) X-gal staining is present in the hair follicle and follicular cells (*) surrounding the hair shaft in Ildr1-/- mice. The nuclei are counterstained with nuclear fast red. Scale bar is 20 μm and scale is the same for panels B and D. (TIF) [file pone.0270329.s004.tif]

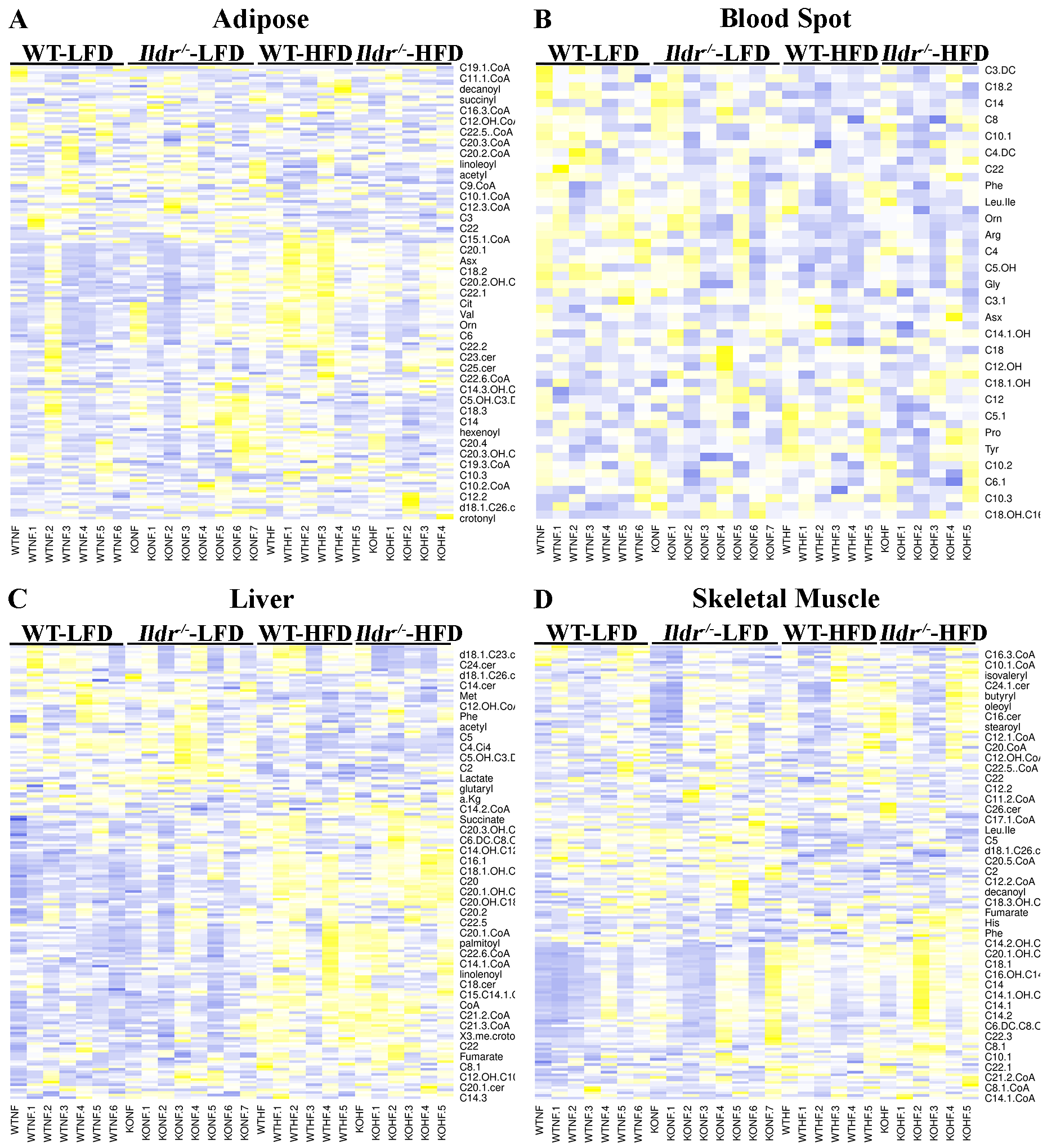

Supplement: S6 Fig — (A) Heat map displaying effects of diet and genotype on the metabolite profiles in adipose tissue. (B) Heat maps displaying effects of diet and genotype on the metabolite profiles in blood. (C) Heat map displaying effects of diet and genotype on the metabolite profiles in liver. (D) Heat map displaying effects of diet and genotype on the metabolite profiles in gastrocnemius muscle. (TIF) [file pone.0270329.s006.tif]
